# Genome-wide identification of the MADS-box transcription factor family in autotetraploid cultivated alfalfa (*Medicago sativa* L.) and expression analysis under abiotic stress

**DOI:** 10.1186/s12864-021-07911-9

**Published:** 2021-08-07

**Authors:** Xueming Dong, Hao Deng, Wenxue Ma, Qiang Zhou, Zhipeng Liu

**Affiliations:** State Key Laboratory of Grassland Agro-ecosystems, Key Laboratory of Grassland Livestock Industry Innovation, Ministry of Agriculture and Rural Affairs, Engineering Research Center of Grassland Industry, Ministry of Education, College of Pastoral Agriculture Science and Technology, Lanzhou University, 730000 Lanzhou, People’s Republic of China

**Keywords:** Abiotic stress, Autotetraploid, Cultivated alfalfa, Expression profiles, *MADS-box* genes, Transcription factor

## Abstract

**Background:**

Alfalfa, the “queen of forage”, is the most extensively cultivated forage legume in the world. The development and yield of alfalfa are seriously limited by abiotic stress. MADS-box transcription factors are one of the largest gene families and play a pivotal role in plant development and abiotic stress. However, little is known regarding the MADS-box transcription factors in autotetraploid cultivated alfalfa.

**Results:**

In the present study, we identified 120 *MsMADS-box* genes in the alfalfa genome. Phylogenetic analysis indicated that 75 type-I *MsMADS-box* genes were classified into the Mα, Mβ, and Mγ subgroups, and 45 type-II *MsMADS-box* genes were classified into 11 subgroups. The promoter region of *MsMADS-box* genes containing several hormone and stress related elements. Chromosomal location analysis revealed that 117 *MsMADS-box* genes were unevenly distributed on 32 chromosomes, and the remaining three genes were located on unmapped scaffolds. A total of nine pairs of segmental duplications and four groups of tandem duplications were found. Expression analysis showed that *MsMADS-box* genes were differentially expressed in various tissues and under abiotic stresses. qRT-PCR analysis revealed that the expression profiles of eight selected *MsMADS-box* genes were distinct under various stresses.

**Conclusions:**

In this study, *MsMADS-box* genes were identified in the cultivated alfalfa genome based on autotetraploid level, and further confirmed by Gene Ontology (GO) analysis, phylogenetic analysis, sequence features and expression analysis. Taken together, these findings will provide clues for further study of *MsMADS-box* functions and alfalfa molecular breeding.

Our study is the first to systematically identify and characterize the MADS-box transcription factors in autotetraploid cultivated alfalfa (*Medicago sativa* L.), and eight *MsMADS-box* genes were significantly involved in response to various stresses.

**Supplementary Information:**

The online version contains supplementary material available at 10.1186/s12864-021-07911-9.

## Background

Transcription factors (TFs) regulate gene expression at the transcriptional level and are involved extensively in plant growth and development, organ morphogenesis, stress and hormone signal responses [1]. The *MADS-box* gene family is one of the largest families of TFs and is widely distributed in eukaryotes. The word “MADS” was derived from the initials of four proteins: *Mini Chromosome Maintenance 1*(*MCM1*) of yeast [2], *Agamous* (*AG*) of *Arabidopsis thaliana* [3], *Deficiens* (*DEF*) of *snapdragon* [4], and *Serum Response Factor* (*SRF*) of humans [5]. According to evolutionary relationships, the MADS-box protein family is clustered into type-I and type-II groups, which might be derived from gene duplication events of the same ancestor [6]. Type-I MADS-box proteins typically contain a highly conserved SRF-like domain with one to two exons and can be further divided into three subgroups: Mα, Mβ, and Mγ [7]. Type-II *MADS-box* genes are also called MIKC-type genes, which consist of two subgroups: MIKC^*^ and MIKC^c^ [8]. According to differences in gene function and sequence homology, MIKC^c^-type genes are classified into 14 subgroups, which are defined as AG, SEP/AGL2, AGL6, AGL12, AGL15, AGL17, FLC, SQUA, TM3/SOC1, TM8, STMADS11, GGM13, and DEF/GLO [9]. Notably, *MADS-box* genes belonging to the same subfamily often show analogous expression patterns and related functions [10, 11].

The *MADS-box* gene family plays not only a regulatory role in the development of flower organs but also important roles in controlling flowering time, determining the differentiation of meristematic tissue, controlling embryonic development, promoting root formation, and regulating the development of seeds and fruits [12, 13]. Recently, many studies have proven that *MADS-box* genes play a significant role in the regulation of plant tolerance to extreme conditions, such as drought, salt, high temperature and cold stress [14, 15]. In rice, knocking down the expression of *OsMADS26* enhances the resistance of rice to drought and pathogenic bacterial stress [16, 17]. In wheat, overexpression of *TaMADS*, a type-II (MIKC) *MADS-box* gene, results in early flowering through upregulation of *LUMINIDEPENDENS* (*LD*) and *FLOWERING CONTROL LOCUS A* (*FCA*) expression under cold treatment [18]. Similarly, overexpression of *SlMBP11*, an AGL15-like gene isolated from *Solanum lycopersicum*, enhances salt tolerance through an abscisic acid-independent signaling network in tomato [19]. Thus, *MADS-box* TFs have major significance in enhancing plant tolerance to various stresses.

Alfalfa (*Medicago sativa* L.) is widely cultivated in North America, Asia and other continents, with an area of more than 40 million hectares [20]. In the Netherlands, two-thirds of arable land has been used for alfalfa to develop grassland agriculture. Alfalfa is the fourth largest planted crop in the United States, after wheat, corn and soybean [21]. In China, alfalfa is planted in 14 provinces throughout the Northeast, Central North and Northwest regions of the country. An excellent local cultivated alfalfa cultivar (‘XinJiangDaYe’) with large leaves and an autotetraploid genome (2n = 4x = 32) is widely planted in Xinjiang, Gansu, Qinghai and other provinces in China and has the highest resistance. Alfalfa has a high biomass and crude protein content and is rich in digestible nutrients and mineral elements, which greatly reduces the cost of feed supplementation for livestock production. Alfalfa is one of the key grasses for promoting agricultural production and economic development in arid and semiarid areas of China [22]. With the deterioration of the ecological environment in these areas, the development and quality of alfalfa are limited, and production is reduced by at least 10–20 % due to multiple extreme conditions such as deficient water, freezing temperatures, and high salinity [23]. In 2020, the complete genome data of the autotetraploid cultivar XinJiangDaYe were published, generating a chromosome-level genome assembly with 32 chromosomes and high quality rather than eight chromosomes [21, 24]. This assembly will provide a wide range of data resources for selecting key stress-related genes and improving alfalfa stress resistance by genetic engineering.

**Fig. 1:**
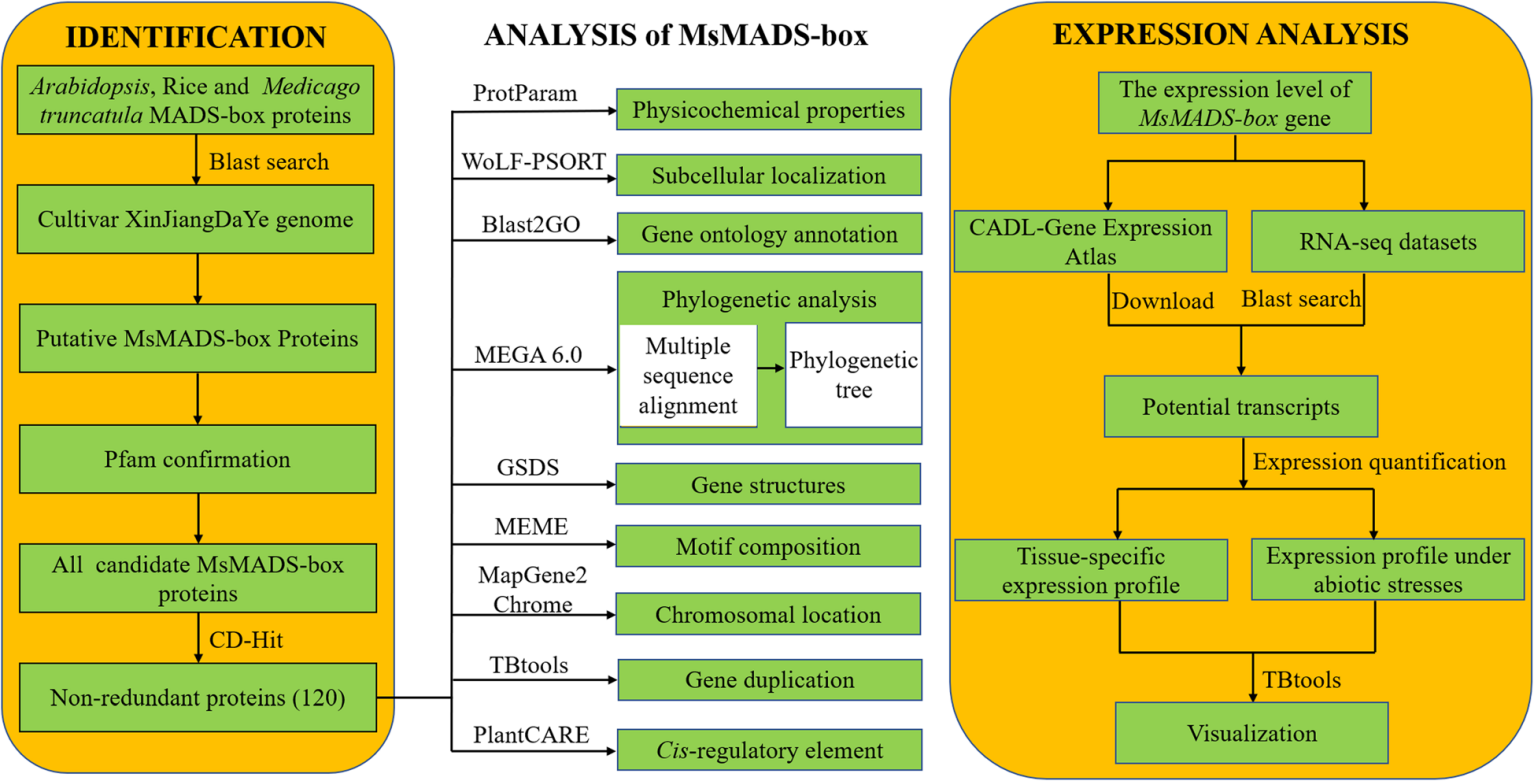
The framework of content in this study

To date, the functions of the *MADS-box* gene family involved in abiotic stress have been widely reported in a variety of plants [25]. Large quantities of *MADS-box* genes have been identified and characterized in *Oryza sativa* (*n* = 75) [26], *Pyrus* × *bretschneideri* (*n* = 95) [27], *A. thaliana* (*n* = 109) [28], *Zea mays* L. (*n* = 142) [29], and *Brassica rapa* (*n* = 167) [30]. However, there have been no reports in terms of *MADS-box* genes involved in stress resistance in alfalfa. Cultivated alfalfa genomic data have been published, and a large amount of transcriptome data are available, providing a reliable experimental resource for the systematic study of *MADS-box* genes in alfalfa [31–33]. Based on these data, in this study, *MADS-box* genes in the genome of the autotetraploid cultivar XinJiangDaYe were identified and characterized. Detailed information on the physicochemical properties, phylogenetic relationships, gene structures, motif composition, chromosomal map, synteny and *cis*-regulatory elements of *MsMADS-box* genes was generated. In addition, the tissue-specific expression patterns and the differential expression of *MsMADS-box* genes under various abiotic stresses were comprehensively surveyed. The framework of content in this study was shown in Fig. 1. Our study is the first to report on the *MADS-box* gene family in autotetraploid cultivated alfalfa, which provides valuable information for exploring the functions and molecular mechanisms underlying abiotic stress tolerance in this important economical crop.

## Results

### Identification and Gene Ontology (GO) analysis of *MsMADS-box* genes in alfalfa

A total of 120 *MADS-box* genes were identified in the alfalfa genome after removal of redundant sequences and were renamed from *MsMADS001* to *MsMADS120* according to their order of appearance in the genome annotation file. Information on MsMADS-box protein sequences and gene sequences is provided in Additional file 1: Table S1. The physicochemical properties of MsMADS-box proteins were investigated by Expasy-ProtParam, and detailed information is summarized in Additional file 1: Table S2. The lengths of the identified MsMADS-box proteins ranged from 95 to 1288 amino acids (AAs). The molecular weights (MWs) of the MADS-box proteins ranged from 11588.13 to 147858.01 Da, and the pIs ranged from 4.69 to 9.87. The instability indices of most MsMADS-box proteins (98 of 120) were higher than 40, which suggested that they were not stable proteins. The grand average of hydropathicity of all MsMADS-box proteins was negative (< 0), indicating that they were soluble hydrophilic proteins. Moreover, the subcellular localization analysis of the MADS-box proteins showed that most of them were located in the nucleus, followed by the mitochondrial matrix space and the plasma membrane.

To figure out the functional classification of all *MsMADS-box* genes, GO annotation was analyzed by Blast2GO 5.2 software. All the *MsMADS-box* genes were categorized into biological process, molecular function, and cellular component categories, and the detail information was summarized in Table 1 and Additional file 1: Table S3. For the biological process category, four terms were assigned to all *MsMADS-box* genes, including “regulation of biological process”, “metabolic process”, “biological regulation”, and “cellular process”, and 108 *MsMADS-box* genes were assigned to “positive regulation of biological process”. Regarding the molecular function category, totals of 119 and 120 *MsMADS-box* genes were assigned to “binding” and “transcription regulator activity” terms, respectively. All *MsMADS-box* genes were classified as “cellular anatomical entity” term, which was the only one term identified in the cellular component category.

**Table 1:** Gene ontology (GO) analysis of 120 *MsMADS-box* genes

GO term	Ontology	Description	Gene number
GO:0050789	Biological Process	regulation of biological process	120
GO:0048518	Biological Process	positive regulation of biological process	108
GO:0008152	Biological Process	metabolic process	120
GO:0065007	Biological Process	biological regulation	120
GO:0009987	Biological Process	cellular process	120
GO:0005488	Molecular Function	binding	119
GO:0140110	Molecular Function	transcription regulator activity	120
GO:0110165	Cellular Component	cellular anatomical entity	120

### Phylogenetic analysis, gene structures and motif composition of *MsMADS-box* genes

**Fig. 2:**
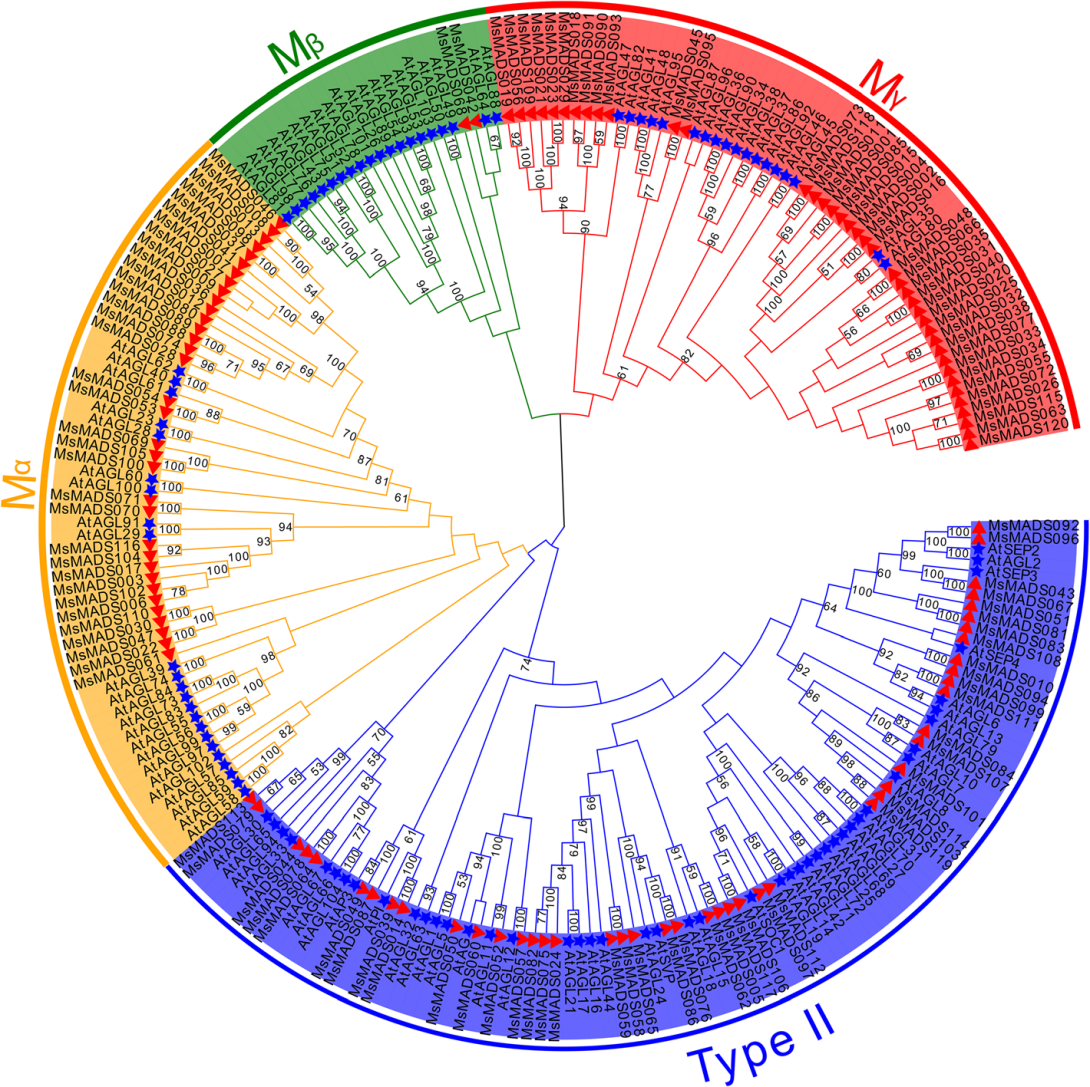
Phylogenetic trees of alfalfa and *Arabidopsis* MADS-box proteins constructed in MEGA-X using the neighbor-joining method. The tree is divided into four groups, indicated by different colors. The blue star and red triangle shapes represent *Arabidopsis* and alfalfa *MADS-box* genes, respectively

To examine the evolutionary relationships among MsMADS-box proteins, a phylogenetic tree was constructed between alfalfa and *Arabidopsis* MADS-box proteins by MEGA-X using the NJ method. The family of 75 type-I *MsMADS-box* genes was divided into the Mα, Mβ, and Mγ subgroups and the 45 type-II *MsMADS-box* genes were divided into 13 subgroups (MIKC^*^, GGM13, AGL17, STMADS11, AGL15, AG, AGL12, DEF + GLO, TM3, FLC, SQUA, AGL6, and AGL2) according to the classification of *MADS-box* genes in *Arabidopsis* (Fig. 2 and Additional file 2: Fig. S1). However, subgroups GGM13 and FLC had no *MsMADS-box* members, subgroup AGL12 contained the smallest number of MsMADS-box proteins (only one), and subgroup Mγ contained the largest number.

Gene structure analysis showed that the number of exons in *MsMADS-box* genes varied from one to 17 (Additional file 2: Fig. S2). Among the 120 *MsMADS-box* genes, *MsMADS117* possessed the highest number (*n* = 17) of exons, followed by *MsMADS059* and *MsMADS062* (*n* = 16) and *MsMADS144* (*n* = 15). Moreover, a total of 62 *MsMADS-box* genes had only one exon, and the number of introns varied among different subfamilies. The type-II *MsMADS-box* genes contained multiple introns, while most type-I *MsMADS-box* genes had zero to two introns, except for *MsMADS042* and *MsMADS068* from the Mβ subfamily, which contained four and five introns, respectively, and *MsMADS090* from the Mα subfamily, which contained six introns.

To explore the distribution and structural diversification of conserved motifs of alfalfa MsMADS-box proteins, the MEME tool was applied to identify conserved motifs, most of which played significant roles in protein-protein interactions and transcriptional activity. A total of 20 conserved motifs were identified and renamed motifs 01 to 20, some of which exhibited similar compositions and positions in the same subfamilies of *MsMADS-box* genes (Additional file 1: Table S4 and Additional file 2: Fig. S3). According to the NCBI CDD (https://www.ncbi.nlm.nih.gov/Structure/cdd/wrpsb.cgi) search results, motif 01 and motif 04 were recognized as the MADS domain and the K domain, respectively. Motif 01, a highly conserved motif, consisted of approximately 60 amino acids in the N-terminus of type-I MsMADS-box proteins, except for *MsMADS04*, *MsMADS027*, *MsMADS033*, *MsMADS034*, and *MsMADS068*. Motif 04, which plays a significant role in protein-protein interactions in *MADS-box* gene family, appeared in all type-II *MsMADS-box* genes.

### Chromosomal mapping and synteny analysis of *MsMADS-box* genes in alfalfa

**Fig. 3:**
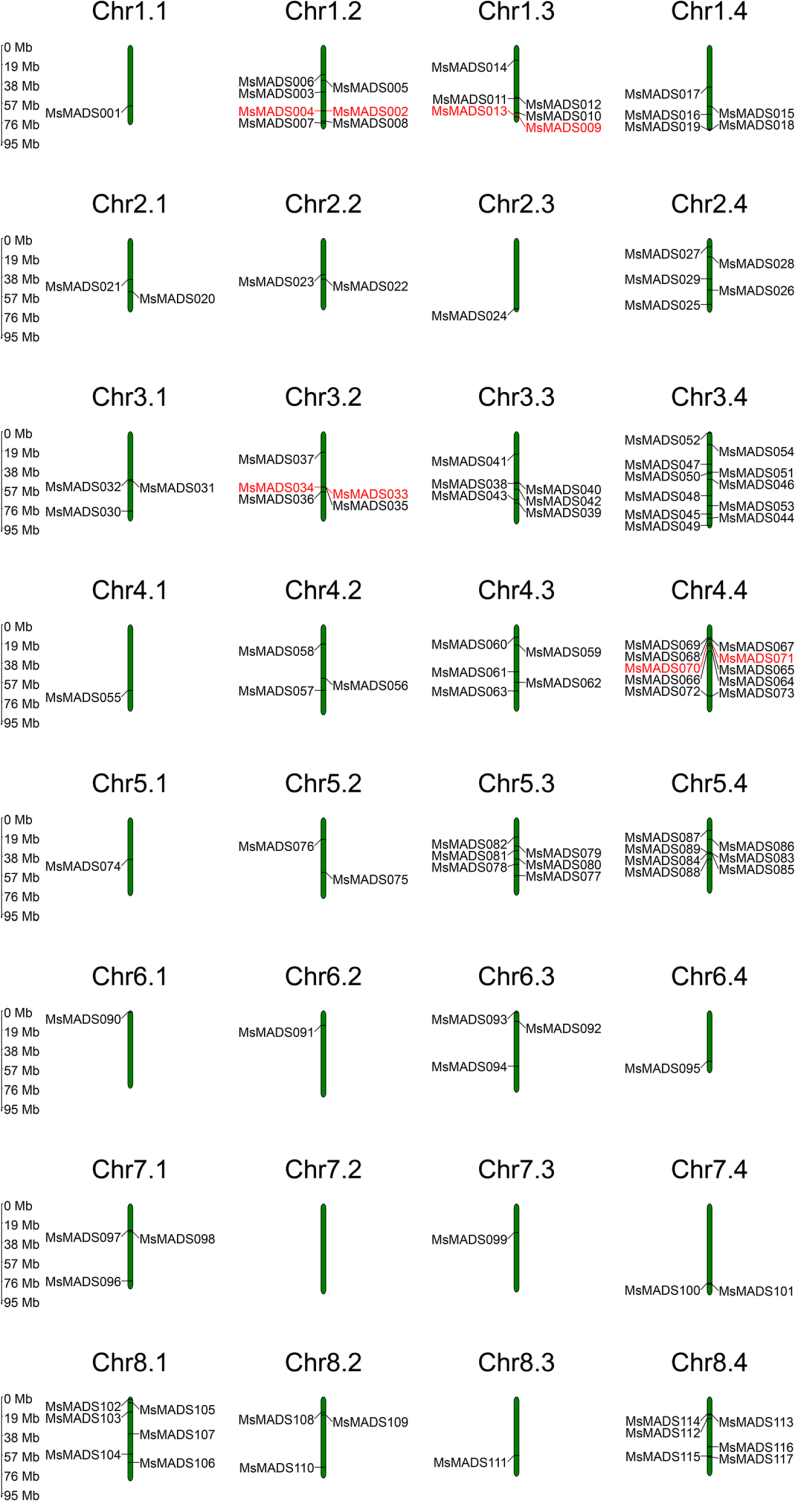
Distribution and position of the *MsMADS-box* genes across the 32 chromosomes in the alfalfa genome. Chr 1.1 to Chr 8.4 represent the linkage groups of alfalfa. The green bars represent each chromosome, and black lines indicate the position of each *MsMADS-box* gene. Red lines represent the tandemly duplicated genes

All *MsMADS-box* genes were unevenly distributed on the 32 chromosomes of alfalfa, except for *MsMADS118*, *MsMADS119*, and *MsMADS120* (Fig. 3). Chromosome 3.4 (Chr 3.4) contained the largest number of *MADS-box* genes (*n* = 11), Chr 4.4 contained 10 members, Chr 5.4 and Chr 1.2 each had 7 members, and Chr 7.2 had no *MsMADS-box* genes. In addition, there were six *MsMADS-box* genes located on Chr 1.3, Chr 3.3, Chr 5.3, Chr 8.1, and Chr 8.4, and five *MsMADS-box* genes were found on Chr 1.4, Chr 2.4, Chr 3.2, and Chr 4.3. Only one *MsMADS-box* gene was distributed on Chr 1.1, Chr 2.3, Chr 4.1, Chr 5.1, Chr 6.1, Chr 6.2, Chr 6.4, Chr 7.3, and Chr 8.3.

**Fig. 4:**
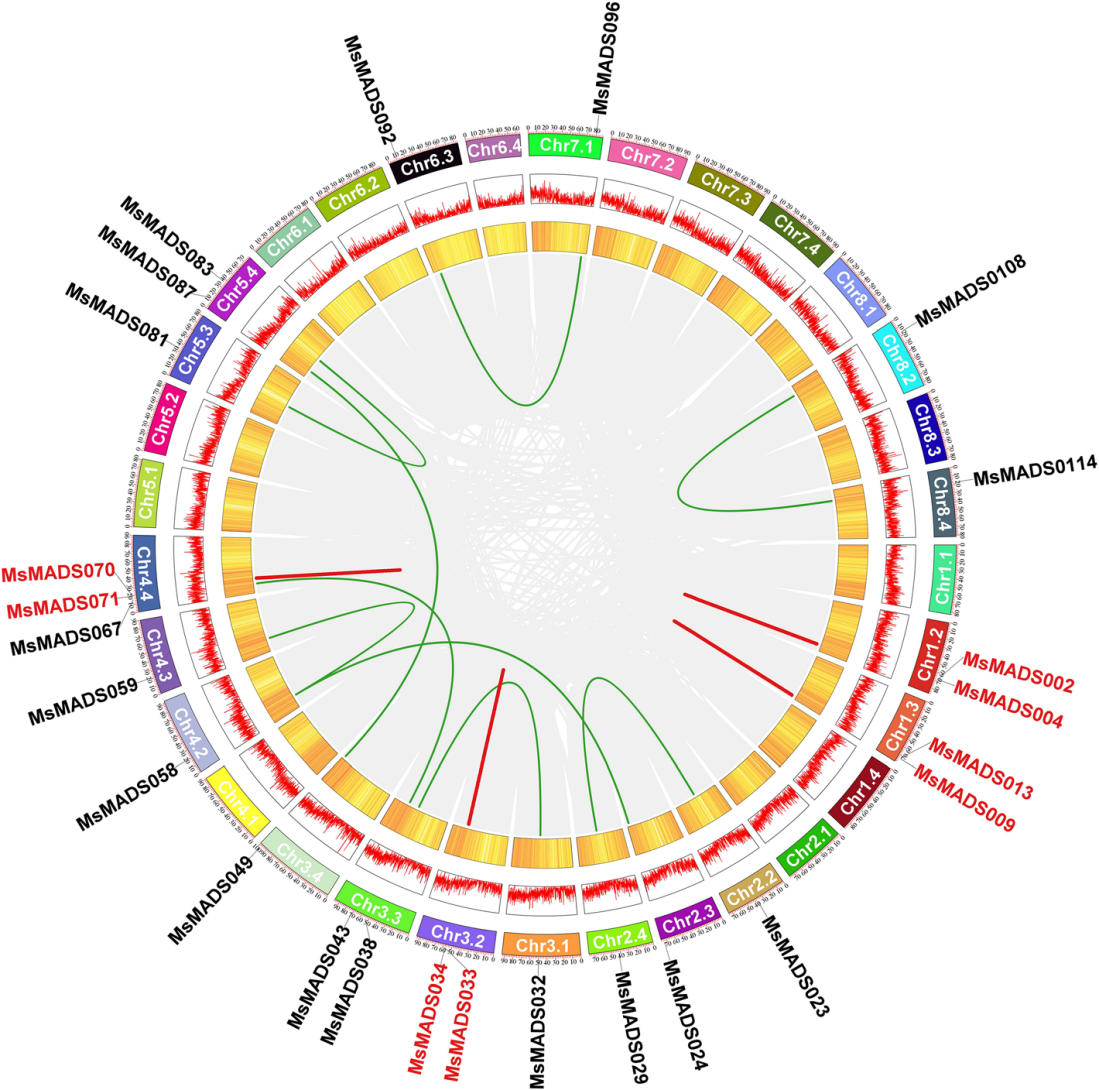
Synteny analysis of *MsMADS-box* genes in alfalfa. Gray lines represent all synteny blocks in the alfalfa genome. Green and red lines indicate the duplicated *MsMADS-box* gene pairs and tandemly duplicated *MsMADS-box* genes in alfalfa, respectively. Yellow and red boxes indicate the gene density and expression levels of *MsMADS-box* genes, respectively

We used TBtools software to perform collinearity analysis to detect the gene duplication events of *MADS-box* genes in alfalfa. A total of nine pairs of duplicated segments in *MsMADS-box* genes and four groups of tandemly duplicated *MsMADS-box* genes (*MsMADS002/MsMADS004*, *MsMADS009/MsMADS013*, *MsMADS033/MsMADS034*, and *MsMADS070/MsMADS071*) were identified (Fig. 4).

### *Cis*-regulatory element analysis of *MsMADS-box* gene promoters

*Cis*-regulatory elements are specific DNA sequences that are located upstream of gene coding sequences and regulate the expression of stress-responsive genes by binding with TFs. Thus, we explored the distribution of six *cis*-regulatory elements in the promoter regions of these changed *MsMADS-box* genes during abiotic stresses. The GT1 motif had the largest number of *cis*-regulatory elements, with 168, followed by ABREs (*n* = 165), MBSs (*n* = 101), CGTCA motifs (*n* = 100), TC-rich repeats (*n* = 63), and LTRs (*n* = 38) (Additional file 2: Fig. S4). Moreover, we found that *MsMADS033*, *MsMADS051*, and *MsMADS075* contained all six *cis*-regulatory elements, while *MsMADS048*, *MsMADS056*, *MsMADS073*, and *MsMADS084* had only one *cis*-regulatory element each.

### Expression pattern analysis of *MsMADS-box* genes in *M. sativa* tissues

**Fig. 5:**
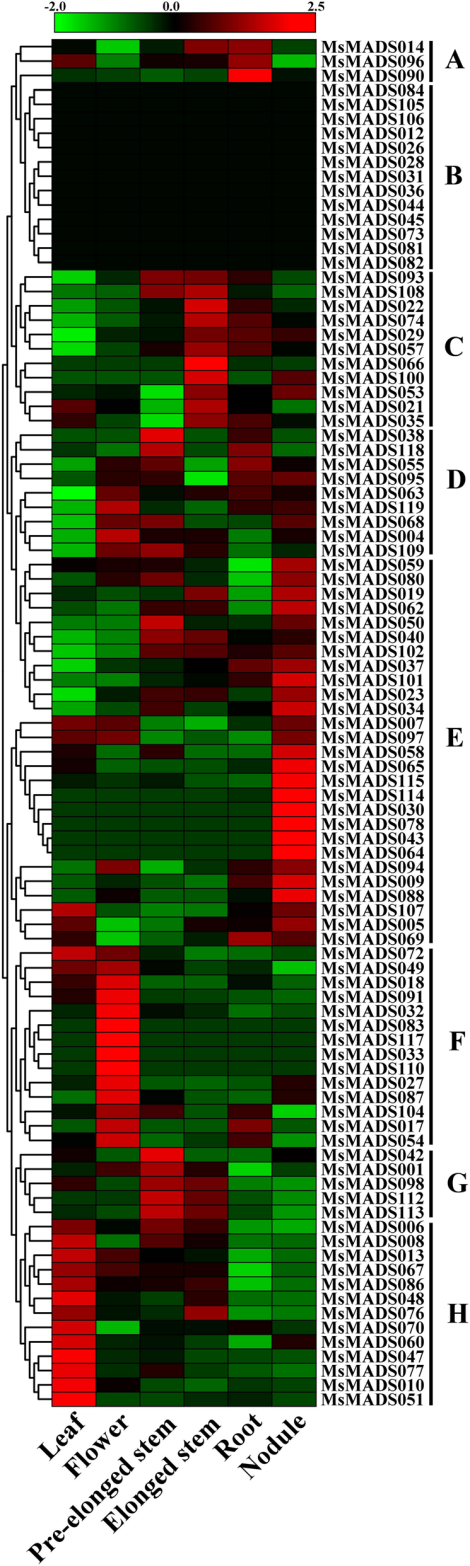
Expression levels of all *MsMADS-box* genes of alfalfa in six tissues (leaf, flower, pre-elongated stem, elongated stem, root, and nodule). The red and green colors indicate the expression levels of *MsMADS-box* genes from high to low, and black indicates the median expression level in the heatmap. Groups A to H exhibited eight expression patterns of *MsMDAS-box* genes in six tissues. The heatmap was generated by using TBtools software

Tissue-specific expression is associated with the specific function of *MADS-box* genes in particular tissues. Thus, we used microarray datasets of the alfalfa B47 genotype obtained from the CADL-Gene Expression Atlas to assess the expression levels of *MsMADS-box* genes in six tissues, including leaf, flower, pre-elongated stem, elongated stem, root, and nodule tissues, of alfalfa. A total of 95 *MsMADS-box* genes were identified, and the remaining 25 genes were not found in the dataset. A heatmap of *MsMADS-box* genes was generated by using TBtools software, and 95 *MsMADS-box* genes were divided into eight groups, namely, A to H, based on their expression levels in different tissues (Fig. 5 and Additional file 1: Table S5). Most of the *MsMADS-box* genes had variable expression profiles, except group B, which was not expressed in any tissues. Groups C, E, F, G, and H showed the highest expression levels in elongated stems, nodules, flowers, pre-elongated stems, and leaves, respectively. Groups A and D exhibited variable expression patterns in these six tissues.

### Expression analysis of *MsMADS-box* genes in response to abiotic stresses

To detect dynamic changes in the expression levels of *MsMADS-box* genes under cold, drought, and salt stresses and ABA treatment, BLASTN against four transcriptome datasets previously collected by our laboratory was performed. A total of 104 *MsMADS-box* genes were identified under these four treatments. A heatmap of these identified *MsMADS-box* genes under cold treatment was separately constructed because the sequenced tissue samples were different from those in the other three treatments. The results are shown in Additional file 1: Table S6 and Additional file 2: Fig. S5. In total, 104 *MsMADS-box* genes were divided into eight groups, namely, groups A to F, according to their expression patterns at different time points under cold treatment. The expression of group A significantly increased at 0 to 2 h and gradually declined at later time points. The expression levels of groups B and D were significantly upregulated under cold stress and peaked at 2 h. Group C did not exhibit expression at any time point under cold treatment. Group E showed downregulated expression at the treatment times. The expression of group F was decreased from 0 to 6 h and increased at 24 h.

For the other three treatments, the expression profiles of 104 *MsMADS-box* genes are shown in Additional file 2: Fig. S6, and the results were similarly classified into groups A to I. For ABA treatment, most *MsMADS-box* genes were either inhibited or not expressed at some time points, such as those in groups A, B, C, D, and E. Group F showed significantly upregulated expression when subjected to ABA treatment. The expression levels of groups G, H and I were significantly upregulated at 3 h and reached their peak values at 12 h.

Under drought treatment, most of the genes showed significant upregulation when subjected to drought treatment and reached their peak values at 1 h, such as those in groups B and D, which indicated that these genes can quickly respond to drought stress. The expression levels of groups A, E, and H were significantly upregulated at 6 h and declined at later time points. The expression levels of groups F and G gradually increased under exposure to drought stress from 1 to 12 h, suggesting positive regulation. The expression of group I was inhibited or repressed from 1 to 24 h, which was different from that in the other groups. Notably, almost all groups showed the minimum relative expression or recovered to the initial transcription level upon drought removal for 1 or 12 h.

Salt treatment also led to serious increases in the transcription levels of groups A, B, C, and D at 1 h, similar to the expression profiles under drought stress. The expression levels of groups E, F, and G reached the minimum upon salt removal for 12 h. The expression pattern of group H was distinct from those of the other groups, which exhibited a dynamic change during salt treatment. For group I, in which expression gradually increased, the peak value appeared at 12 h of salt removal.

### qRT-PCR validation of gene expression

**Fig. 6:**
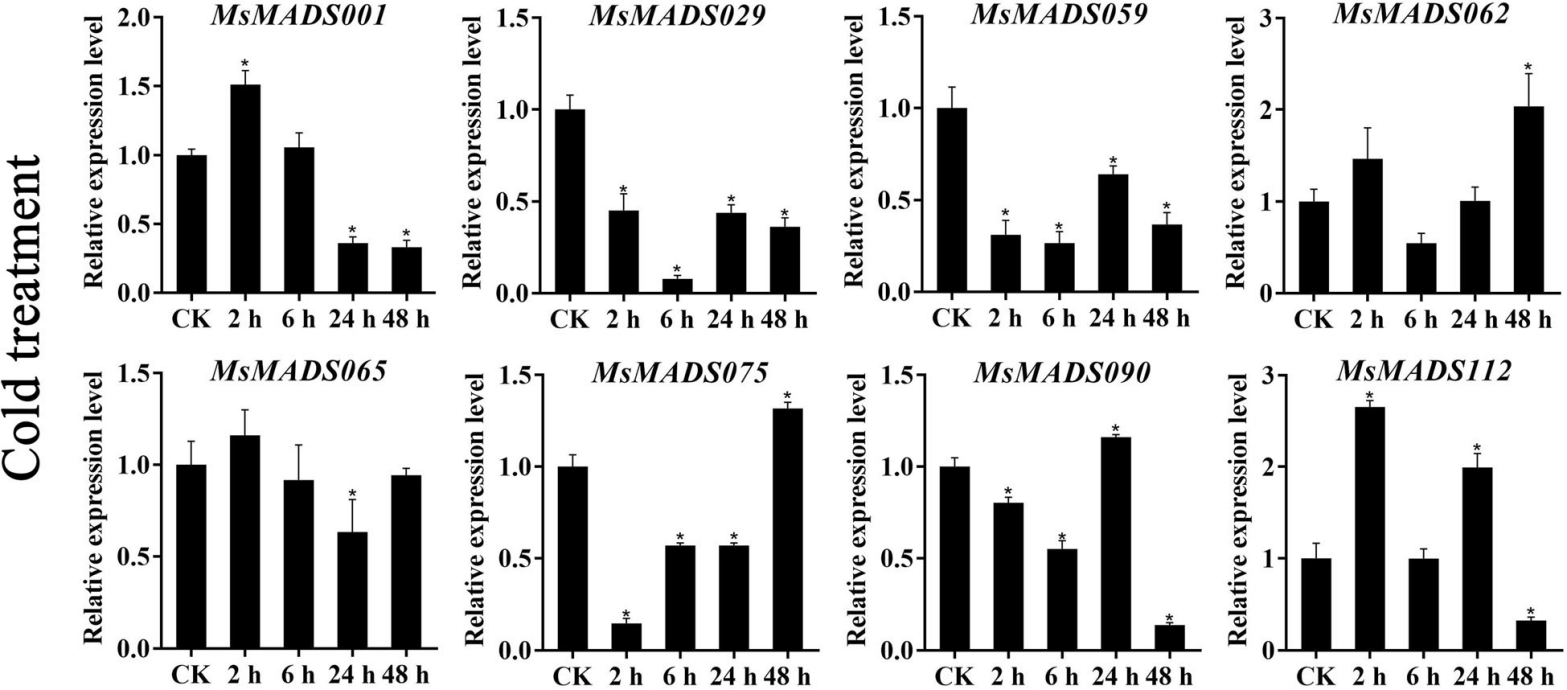
Gene expression analysis of eight *MsMADS-box* genes under cold treatment for 0, 2, 6, 24, and 48 h using qRT-PCR. The error bars indicate the standard errors of three biological replicates. Asterisks represent significant differences compared with “CK”, and *P* < 0.05 (^*^) was considered highly significant

**Fig. 7:**
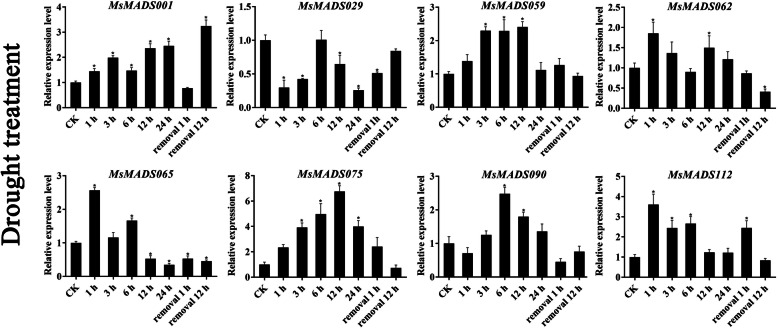
Gene expression analysis of eight *MsMADS-box* genes under drought treatment for 0, 1, 3, 6, 12, and 24 and 1 and 12 h after removal using qRT-PCR. The error bars indicate the standard errors of three biological replicates. Asterisks represent significant differences compared with “CK”, and *P* < 0.05 (^*^) was considered highly significant

**Fig. 8:**
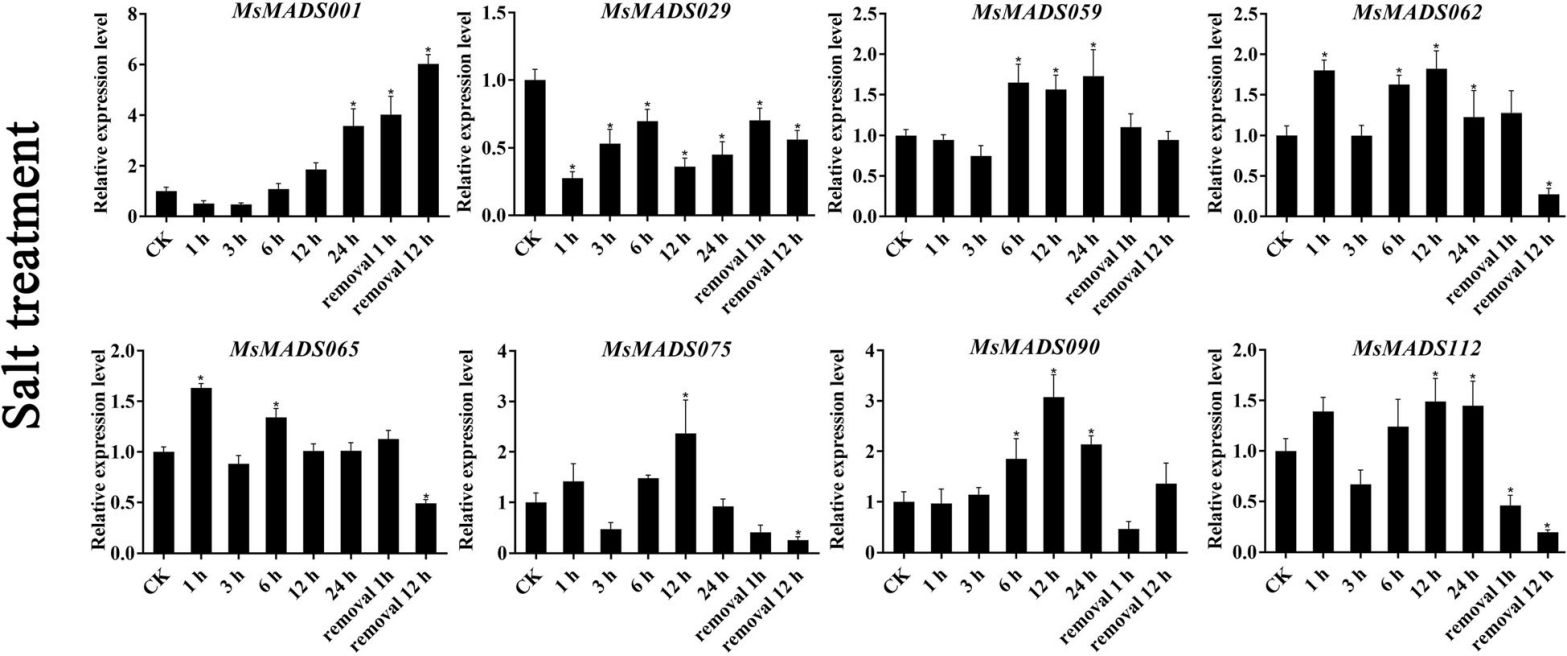
Gene expression analysis of eight *MsMADS-box* genes under salt treatment for 0, 1, 3, 6, 12, and 24 and 1 and 12 h after removal using qRT-PCR. The error bars indicate the standard errors of three biological replicates. Asterisks represent significant differences compared with “CK”, and *P* < 0.05(^*^) was considered highly significant

**Fig. 9:**
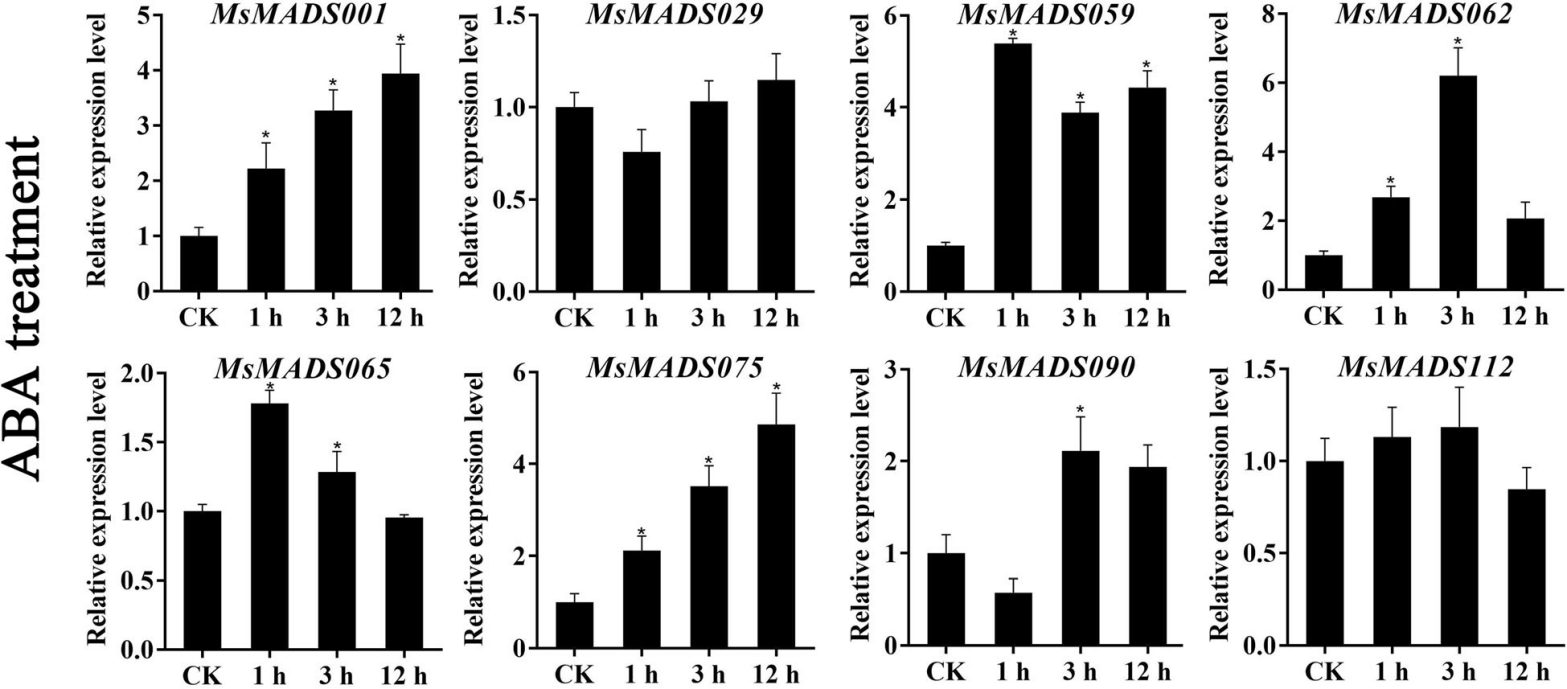
Gene expression analysis of eight *MsMADS-box* genes under ABA treatment for 0, 1, 3, and 12 h using qRT-PCR. The error bars indicate the standard errors of three biological replicates. Asterisks represent significant differences compared with “CK”, and *P* < 0.05(^*^) was considered highly significant

To further verify the RNA-Seq data, eight *MsMADS-box* genes (*MsMADS001, 029, 059, 062, 065, 075, 090*, and *112*) that were significantly induced by cold, drought, and salt stresses and ABA treatment were selected for qRT-PCR validation, and the Ct values of qRT-PCR were provided in Additional file 1: Table S7. The expression trends of most of the tested *MsMADS-box* genes were consistent with the RNA-Seq analysis results (Figs. 6, 7 and 8, and 9). Expression of most of the tested genes was upregulated upon exposure to cold, drought, and salt stresses and ABA treatment, indicating positive regulation. For example, the expression levels of *MsMADS001* and *MsMADS075* were gradually upregulated with time under ABA treatment, which was similar to the RNA-Seq analysis results. The expression of the *MsMADS075* gene showed a trend of initially increasing and then decreasing under drought treatment compared with the control in both the RNA-Seq and qRT-PCR results.

In addition, to examine the effect of circadian rhythm for gene expression, we performed qRT-PCR analysis of eight *MsMADS-box* genes in plants grown without treatments and these results were shown in Additional file 2: Figs. S7, S8 and S9. we found that the expression level of few *MsMADS-box* genes showed circadian rhythm, and the fold change of expression level was not significant. For example, in the absence of drought and salt treatments, the expression level of *MsMADS001* decreased initially, followed by an increased, but then again decreased, while the expression level of *MsMADS001* exhibited a trend of gradual increase, and showed a significant difference compared with CK group under drought and salt treatments. Moreover, after ABA treatment, the expression trend of *MsMADS001* was opposite to that of untreated, indicating that the expression of *MsMADS001* was mainly affected by abiotic stress rather than circadian rhythm. It is worth noting that the expression of *MsMADS029* showed a similar fold change and pattern between salt treatment and without salt treatment, suggesting that the expression of *MsMADS029* may be mainly affected by circadian rhythm rather than stress. Similarly, the expression pattern of *MsMADS065* under cold treatment was similar to without cold treatment, indicating that *MsMADS065* was also mainly related to circadian rhythm.

## Discussion

MADS-box TFs are one of the most widely studied superfamilies in plants. To date, most studies on MADS-box function have focused on the regulation of plant vegetative and reproductive development, floral organogenesis and flowering time [34]. Some MADS-box TFs involved in stress-responsive processes have been found in many plants [19]. For example, many MADS-box proteins in wheat were induced under abiotic stress [15], and *CaMADS* isolated from pepper was also rapidly upregulated by high temperature and salt stress as well as some plant exogenous hormones [35]. However, there have been no studies on the molecular function of MADS-box TFs in response to abiotic stress in alfalfa. Therefore, this study was focused on systematically exploring the potential molecular functions of *MsMADS-box* genes. First, we investigated the features of *MsMADS-box* genes by using genome-wide identification. Moreover, the gene structure, motif composition, phylogenetic relationships, chromosomal location, synteny, *cis*-regulatory elements, tissue-specific expression and responses to cold, drought, and salt stresses and ABA treatment were investigated. These results will enrich our understanding of the *MsMADS-box* gene family and provide a foundation for further exploring the functions of *MsMADS-box* genes.

In our study, a total of 120 *MsMADS-box* genes were identified in the alfalfa genome. The number of *MADS-box* genes in alfalfa was higher than those in *A. thaliana* (*n* = 109) [28], *Oryza sativa* (*n* = 75) [26], *Lactuca sativa* (*n* = 82) [36], *Erigeron breviscapus* (*n* = 44) [37], and *Phyllostachys edulis* (*n* = 42) [38] but lower than those in tomato (*n* = 131) [39], *Zea mays* (*n* = 142) [29], *Brassica rapa* (*n* = 167) [30], and *Gossypium hirsutum* (*n* = 207) [25]. The nucleotide sequence lengths, molecular weights and theoretical isoelectric points of the *MsMADS-box* genes varied. GO annotation analysis indicated that most of *MsMADS-box* genes were related to “regulation of biological process”, “positive regulation of biological process”, “metabolic process”, “biological regulation”, and “cellular process”, which was consistent with the findings in *Helianthus tuberosus* L. [40]. In addition, many GO terms were also assigned to “Molecular Function” and “Cellular Component”, including “binding”, “transcription regulator activity”, and “cellular anatomical entity”, which suggesting that *MsMDAS-box* genes are involved in various biological processes. The gene structure and motif composition were conserved in the same subgroups, indicating that these genes may have subgroup-specific functions [39]. Phylogenetic analysis revealed that 75 *MsMDAS-box* genes belong to type-I genes, which was higher than the numbers in *Arabidopsis* and rice, suggesting that type-I genes have a higher duplication rate during evolution [41]. In contrast to those in *A. thaliana* and rice, the alfalfa *MADS-box* gene family lost the GGM13 subgroup, which is involved in seed pigmentation and endothelial cell development [42]. This difference may be due to interspecific differences or other undetermined causes.

It is well known that gene duplication plays an important role in the rapid expansion and evolution of gene families [27]. There were nine pairs of segmental duplications and four pairs of tandem duplications in the *MsMADS-box* genes. Duplication of *MADS-box* genes has been found in many species, including *Arabidopsis thaliana*, rice and tomato [39]. Chromosome location analysis showed that the *MsMADS-box* genes were unevenly distributed on 32 chromosomes. Moreover, the *MADS-box* gene family in alfalfa was found to have a gene cluster on Chr 3.4, which indicated that the *MsMADS-box* gene family was characterized by a gene duplication phenomenon. Ren et al. identified 110 MIKC-subgroup genes in *Gossypium hirsutum*, which were distributed on 25 chromosomes, and no MIKC-subgroup genes were distributed on chromosome D01 [43]. In our study, 45 MIKC-subgroup genes were identified in the alfalfa genome, and no MIKC genes were found on many chromosomes, suggesting that the differences in mapping results may be related to the reference genomes used. The promoter regions of *MADS-box* gene family members in alfalfa contained a variety of response elements, mainly includes hormone-related elements and stress-related elements, among which the GT1 motif elements were the most abundant, followed by ABREs. This result was similar to the experimental discovery of *MADS-box* genes in Eudicots [44]. In fact, a lot of studies have also been verified that common *cis*-regulatory elements also existed in the promoter region of stress-responsive genes, such as *MYB*, *WRKY*, and *Dof* genes [45–47]. Moreover, previous studies have reported that ABREs are the main *cis*-regulatory elements of ABA response genes, which can be activated to improve plant stress tolerance through interacted with upstream TFs [48]. Therefore, these results further suggest the probable function of *MsMADS-bo*x genes in response to abiotic stresses, and further study need to verify their potential roles.

*MADS-box* gene family is the most powerful TFs that regulate fruit development and ripening and have been extensively studied in *A. thaliana* [28] and perennial woody plants such as *Malus* x *domestica* [49], *Prunus persica* [50], *Pyrus* × *bretschneideri* [27] and *Morella rubra* [51]. Gene expression profiles provide important information for gene function exploration. As a result, we found that 58/95 *MsMADS-box* genes were expressed in all six examined tissues, while some of the other genes showed tissue-specific expression patterns. For example, *MsMADS033, MsMADS083, MsMADS110*, and *MsMADS117* were expressed only in flowers, suggesting that these genes play key roles in the regulation of floral organogenesis and flowering time. This phenomenon was also observed in rice [52], wheat [53], and soybean [54]. In addition, tissue-specific overexpression of *MADS-box* genes has been reported to regulate flowering time. For example, overexpression of *TaMADS* in *Arabidopsis* leads to early flowering [18], and overexpression of *NtMADS133* in tobacco significantly reduces the flowering time from 58 days to 38 days [55]. Moreover, *MsMADS009, MsMADS057*, and *MsMADS095* were expressed at high levels in all tested tissues (FPKM > 20), suggesting that these genes play important roles throughout the plant life cycle. Therefore, we speculate that *MADS-box* genes with tissue-specific expression have important regulatory functions in related tissues, which provides insight into how we can utilize *MADS-box* genes to promote tissue growth and development.

The related role of MADS-box TFs in plant stress resistance has been reported in earlier studies in various plants, such as rice [56], tomato [14], sheepgrass [57], *Brassica rapa* [30], and wheat [15]. For example, in maize, the expression of *ZZM7-L*, an AGL12-like *MADS-box* gene, was upregulated by NaCl, cold, and mannitol treatments and downregulated by exogenous ABA, and overexpression of *ZZM7-L* in *Arabidopsis thaliana* resulted in a significantly lower germination rate of the transgenic plants than of the wild type, suggesting that *ZZM7-L* had a negative function [29]. Similarly, the expression of *CaMADS* was induced by low temperature, salt, and ABA, and *CaMADS*-overexpressing *Arabidopsis* plants were more tolerant to these stresses than WT plants [39]. In *Ginkgo biloba*, a recent study also demonstrated that *GbMADS9*, a GGM13-clade MADS-box protein, enhanced the tolerance of transgenic *Arabidopsis* plants to osmotic stress, suggesting that *GbMADS9* had a positive function in response to osmotic stress [58]. In this study, the expression patterns of 104 *MsMADS-box* genes under multiple abiotic stresses were determined in previous RNA-Seq data. Most *MsMADS-box* genes showed different responses to abiotic stress at the transcriptional level. In addition, we found that *MsMADS001*, *MsMADS075* and *MsMADS090* were significantly induced by drought and salt stresses, suggesting that these genes might have multiple stress resistance-related functions in alfalfa. The expression levels of eight *MsMADS-box* genes under cold, drought, and salt stresses and ABA treatment were studied by qRT-PCR. However, there were variations in the degrees of gene expression fold change between the RNA-seq and qRT-PCR analyses, which also appeared in several previous studies [59, 60] and may be due to genetic diversity among alfalfa individuals [31]. Moreover, we performed qRT-PCR experiments on the expression of eight *MsMADS-box* genes at different time points in the plants grown without stresses treatment to investigate whether *MsMADS-box* genes expression was induced by circadian rhythm. These results showed that only small proportion of *MsMADS-box* gene was involved in circadian rhythm, which was consistent with the found in pineapple [61]. In conclusion, the transcriptional responses of *MsMADS-box* genes to cold, drought, and salt stress and ABA treatment will provide a solid foundation for further studies of abiotic stress response mechanisms in alfalfa.

## Conclusions

In this study, we comprehensively and systematically analyzed the MADS-box transcription factor family in the autotetraploid cultivated alfalfa genome. A total of 120 *MsMADS-box* genes were extensively identified via genome-wide screening. The physicochemical properties, phylogenetic relationships, exon-intron structures, conserved motifs, chromosomal location, gene duplication, *cis*-regulatory elements, tissue-specific expression patterns, and expression levels under cold, drought and salt stress and ABA treatment were analyzed. Expression analysis of *MsMADS-box* genes provides insights for further understanding the molecular mechanisms of *MsMADS-box* genes underlying development and responses to abiotic stress in autotetraploid cultivated alfalfa. This study provides clues for understanding the potential molecular function of *MADS-box* gene in alfalfa, and lay a foundation for enhancing alfalfa tolerance to abiotic stresses by overexpressing, RNA interference, or CRISPR/Cas9 technology in the future.

## Methods

### Identification and Gene Ontology (GO) analysis of the *MsMADS-box* gene family in alfalfa

The genome assembly files of alfalfa were downloaded from the figshare projects (https://figshare.com/projects/whole_genome_sequencing_and_assembly_of_Medicago_sativa/66380). To more comprehensively investigate the *MADS-box* family genes, the MADS-box protein sequences of *Arabidopsis* and rice were obtained from The *Arabidopsis* Information Resource (TAIR9) (www.arabidopsis.org) database and the Rice Genome Annotation Project (http://rice.plantbiology.msu.edu/), respectively. Moreover, to further accurately identify the *MADS-box* gene family in the alfalfa genome, the MADS-box protein sequences of *Medicago truncatula*, a model plant for legumes, were acquired from the LIS-Legume Information System (https://legumeinfo.org/home). First, these protein sequences were used as query sequences for BLAST analysis against the cultivar XinJiangDaYe genome database with an E-value cutoff of 0.00001. Furthermore, a hidden Markov model (HMM) profile of *MADS-box* (PF00319) was downloaded from the Pfam database (http://pfam.xfam.org/) for further confirmation of alfalfa MADS-box proteins with HMMER 3.0 software using default parameters, and an E-value of 1.0 was set as the threshold. The CD-HIT web server (http://www.bioinformatics.org/cd-hit/) with default parameters was used to remove redundant data. Subsequently, these purified sequences were considered *MsMADS-box* genes for further analysis. The ProtParam tool (https://web.expasy.org/protparam/) was used to determine the physicochemical properties of each MsMADS-box protein, including the molecular weight (MW), theoretical isoelectric point (pI), instability index and grand average of hydropathicity (GRAVY) index. In addition, the subcellular localization of all *MsMADS-box* genes was predicted by WoLF-PSORT (https://www.genscript.com/wolf-psort.html). In addition, GO annotation was performed to investigate the functional role of *MsMADS-box* genes using Blast2GO 5.2 software, with default parameters [62].

### Phylogenetic analysis, gene structures and motif composition of *MsMADS-box* genes

To analyze the evolutionary relationships of the obtained *MADS-box* genes in alfalfa, multiple sequence alignment was performed by Clustal W (http://www.clustal.org/clustal2/) with default parameters, and a phylogenetic tree of 120 alfalfa *MADS-box* and 107 *Arabidopsis MADS-box* genes was then constructed using the neighbor-joining (NJ) method in MEGA 6.0 software (https://www.megasoftware.net/) with a p-distance model, 1000 bootstrap replicates, and pairwise detection. All *MsMADS-box* genes were classified into the Mα, Mβ and Mγ subgroups and type-II groups according to their evolutionary relationships with *MADS-box* genes in *Arabidopsis*.

Understanding the gene structure can help reveal gene functions, regulation and evolution. To investigate the diversity of gene structures of *MADS-box* genes, the Gene Structure Display Server (GSDS) (http://gsds.cbi.pku.edu.cn) program was applied. The MADS domain is the core of MADS-box TFs, which can activate the downstream genes through interact with the promoter of them. The MEME 4.12.0 online tool (http://meme-suite.org) was used to identify the conserved motifs of these 120 alfalfa MADS-box proteins. The parameters were set as follows: the maximum number of motifs was 20; zero or one occurrence per sequence (zoops) was selected as the site distribution; the minimum and maximum motif widths were set to 6 and 200, respectively; and other parameters were set to the default values.

### Chromosomal mapping and gene duplication analysis

In order to better recognize the genomic distribution of *MsMADS-box* genes, MapGene2Chrome (http://mg2c.iask.in/mg2c_v2.0/) was used to draft a chromosomal location map of the *MADS-box* genes based on the genome annotation files of alfalfa. Gene duplication is a major source to produce new genes and lead to the proliferation and diversification of TFs genes in the plant kingdom. TBtools software was used to perform a collinearity analysis of 120 *MsMADS-box* genes in order to detect gene duplication events [63].

### *Cis*-regulatory element analysis

In-depth analysis of the *cis*-regulatory elements contained in *MsMADS-box* genes will provide further insight into their roles in plant stress resistance. The PlantCARE database (http://bioinformatics.psb.ugent.be/webtools/plantcare/html/) was used to identify the *cis*-regulatory elements within the sequence 2000 bp upstream of transcriptional initiation sites of *MsMADS-box* genes whose expression changed under abiotic stresses. A total of six *cis*-regulatory elements known to be associated with stress and hormones have been identified in the promoter regions of *MADS-box* genes in alfalfa, including ABRE, TC-rich repeats, MBS, LTR, GT1-motif, and CGTCA-motif, which are involved in abscisic acid-responsive, defense- and stress-responsive, drought-inducible, low-temperature-responsive, light-responsive, and MeJA-responsive processes, respectively.

### Tissue-specific expression analysis of *MsMADS-box* genes in alfalfa

The expression pattern of the *MsMADA-box* genes in different tissues can infer the role in the process of tissue development of alfalfa. Genome-wide transcriptome data were used to explore the expression profiles of *MADS-box* genes in different tissues during the development of alfalfa, which were downloaded from the CADL-Gene Expression Atlas (https://www.alfalfatoolbox.org) provided by the Noble Research Institute [64]. The expression data of six tissues, including leaves, flowers, pre-elongated stems, elongated stems, roots, and nodules, were analyzed. TBtools software was used to cluster these expression data and generate a heatmap.

### Expression analysis of *MsMADS-box* genes under abiotic stresses

In previous studies, to acquire additional genetic information on alfalfa under abiotic stresses, four RNA-Seq projects of alfalfa under cold (SRR7091780-SRR7091794), drought, salt and ABA treatments (SRR7160313-SRR7160357) were performed. In this study, to realize the roles of *MsMADS-box* genes in response to abiotic stresses, the nucleotide sequences of all *MsMADS-box* genes were used as query sequences for local nucleotide blast (BLASTN) against these four transcriptome datasets [65]. Subsequently, TBtools software was applied to calculate the gene expression values and draw a heatmap of *MsMADS-box* genes under abiotic stresses.

### Plant materials and stress treatments

The plant material used in this experiment was cultivated ‘XinJiangDaYe’ alfalfa. All experimental materials were obtained using a hydroponic method in this study. First, we selected seeds that were full and uniform in shape. After alfalfa seeds were surface sterilized, they were regularly placed onto two layers of filter paper moistened with distilled water in square Petri dishes at 22 ℃. After germination for 5 days, seedlings with the same taproot length were uniformly transferred into a hydroponic pot containing 1/2 MS nutrient solution (pH = 5.8) that was changed every two days. Subsequently, the seedlings were cultivated under the following conditions: 16 h light/8 h dark cycle, relative humidity of 80 %, and a temperature of 22 °C. The alfalfa seedlings were subjected to different stress treatments when the third leaf appeared (approximately 10 days after germination). For the cold treatment, alfalfa seedlings were transferred to an artificial climate chamber at 4 ℃ for 0, 2, 6, 24, and 48 h, and then whole seedlings of alfalfa were collected. For exogenous ABA treatment, 10 µΜ ABA dissolved in 1/2 MS nutrient solution was used. The root tips of alfalfa seedlings were collected at 0, 1, 3 and 12 h after ABA treatment. To simulate drought and salt stress conditions, individual alfalfa seedlings were immersed in 1/2 MS nutrient solution containing 400 mM mannitol and 250 mM NaCl, respectively. Subsequently, the root tips of the alfalfa seedlings were harvested after drought and salt stress conditions at different time points (0, 1, 3, 6, 12, and 24 h and 1 and 12 h after stress removal). In order to be consistent with the experimental materials used in the previous transcriptome sequencing in our laboratory, total of six whole seedlings were harvested and mixed into frozen tubes at each time point for cold treatment, and six root tips were collected in the same method for ABA, drought, and salt treatment. All samples were quickly frozen in liquid nitrogen and stored at -80 °C until use.

### Quantitative real-time PCR analysis

Eight *MsMADS-box* genes were selected for qRT-PCR experiments to validate the RNA-Seq data. The TRIzol method (Sangon Biotech, Shanghai, China) was used to isolate the total RNA of the whole seedlings under cold treatment (0, 2, 6, 24, and 48 h) and root tips under drought and salt treatments (0, 1, 3, 6, 12, and 24 h and 1 and 12 h after stress removal) and ABA treatments (0, 1, 3 and 12 h). Subsequently, a NanoDrop ND1000 spectrophotometer (Thermo Scientific, Waltham, MA, USA) was applied to determine the concentration of each sample. The first-strand cDNA of each sample was synthesized using a FastKing RT Kit (Tiangen Biotech, Beijing, China) according to the manufacturer’s instructions. The expression patterns of *MsMADS-box* genes were explored by using a CFX96 Real-Time PCR Detection System (Bio-Rad, Los Angeles, CA, USA) with 2xSG Fast qPCR Master Mix (Sangon Biotech, Shanghai, China). The total reaction system was 10 µL, containing 5 µL of 2xSG Fast qPCR Master Mix, 2.6 µL of ddH_2_O, 1 µL of DAF buffer, 1 µL of cDNA, 0.2 µL of upstream primer, and 0.2 µL of downstream primer. The qRT-PCR protocol was as follows: 95 ℃ for 30 s, followed by 40 cycles of 95 °C for 5 s and 60 °C for 30 s. Primer Premier 6 software (Premier Biosoft International, Palo Alto, CA, USA) was applied to design the specific primers for qRT-PCR, and the primer sequences are listed in Additional file 1: Table S8. The *Medicago actin* gene was used as the reference gene. Three biological replicates of each of the samples for qRT-PCR analysis were analyzed. The relative quantification method (2^−ΔΔCT^) was used to calculate the relative expression levels of *MsMADS-box* genes [66]. In addition, it is known that the expression of few genes exhibits circadian rhythm, which oscillates a rhythm signal with a 24 h cycle. Therefore, we examined the expression pattern of eight *MsMADS-box* genes over time under unstressed conditions to verify the effect of circadian rhythm. For example, in the case of cold treatment, we investigated eight *MsMADS-box* genes expression levels of 0, 2, 6, 24, and 48 h in plants grown without cold treatment. For drought and salt treatments, we explored these genes expression of 0, 1, 3, 6, 12, and 24 h and removal 1 and 12 h in plants grown in water. As for ABA treatment, we surveyed the gene expression of 0, 1, 3 and 12 h in plant grown under without treatment conditions.

## Supplementary Information

**Additional file 1: Table S1.** List of the MADS-bBox sequences in Alfalfa. **Table S2.** Protein property of MsMADS-box proteins. **Table S3.** Gene ontology (GO) annotation results of 120 *MsMADS-box* genes. **Table S4.** The structural features of motif 1-20. **Table S5.** Expression (FPKM) of *MsMADS-box* genes in different tissues. **Table S6.** Expression (FPKM) of *MsMADS-box* genes in response to cold, ABA, drought, and salt treatment in alfalfa. **Table S7.** Ct values of eight *MsMADS-box* genes in response to cold, drought, salt, and ABA treatment in alfalfa. **Table S8.** Sequences of primers used in qRT-PCR.

**Additional file 2: Figure S1.** Phylogenetic tree of Type II *MADS-box *genes in alfalfa and *Arabidopsis* constructed using the NJ method in MEGA-X. Group II is divided into 13 subgroups represented by different colors, and alfalfa and *Arabidopsis MADS-box* genes are indicated by blue stars and red triangles, respectively. **Figure S2.** Gene structure analysis of *MsMADS-box *genes in alfalfa. Black lines represent the introns, and green blocks represent the exons. **Figure S3.** Motif analysis of MsMADS-box proteins. Each motif is represented by boxes of different colors for motifs 01 to 20. **Figure S4.** *Cis*-regulatory elements analysis of the promoter regions of *MADS-box *genes of alfalfa. The differently colored boxes with numbers indicate the numbers of *cis*-regulatory elements in the promoter regions of *MADS-box *genes. **Figure S5.** Expression levels of 104 *MADS-box* genes in response to cold treatment in alfalfa. Heat map showing the changes in the relative expression of these genes at 0 (CK), 2, 6, 24, and 48 h under cold treatment at 4 ℃ in the whole seedling. Groups A to F exhibited six expression patterns of the tested *MADS-box *genes under cold treatment. **Figure S6.** Expression levels of 104 *MADS-box *genes in alfalfa under ABA, drought, and salt treatments. Heatmap showing the relative expression levels of total *MADS-box *genes at different time points after ABA treatment (0, 1, 3 and 12 h), drought treatment (0, 1, 3, 6, 12, and 24 h 1 h and 12 h after removal), and salt treatment (0, 1, 3, 6, 12, and 24 h and 1 h and 12 h after removal) in the root tip; “CK” represents 0 h. Groups A to I show nine expression patterns of *MADS-box *genes under the three treatments. **Figure S7.** Gene expression analysis of eight *MsMADS-box *genes without cold treatment for 0, 2, 6, 24, and 48 h using qRT-PCR. The error bars indicate the standard errors of three biological replicates. Asterisks represent significant differences compared with “CK”, and *P* < 0.05 (^*^) was considered highly significant. **Figure S8.** Gene expression analysis of eight *MsMADS-box *genes without drought and salt treatment for 0, 1, 3, 6, 12, and 24 h and 1 h and 12 h after removal using qRT-PCR. The error bars indicate the standard errors of three biological replicates. Asterisks represent significant differences compared with “CK”, and *P* < 0.05 (^*^) was considered highly significant. **Figure S9.** Gene expression analysis of eight *MsMADS-box* genes without ABA treatment for 0, 1, 3, and 12 h using qRT-PCR. The error bars indicate the standard errors of three biological replicates. Asterisks represent significant differences compared with “CK”, and *P* < 0.05(^*^) was considered highly significant.

## Data Availability

The draft genome data of autotetraploid cultivated alfalfa was obtained from https://figshare.com/projects/whole_genome_sequencing_and_assembly_of_Medicago_sativa/66380. The *Arabidopsis* and rice MADS-box protein sequences were downloaded from The *Arabidopsis* Information Resource (TAIR9) (www.arabidopsis.org) and the Rice Genome Annotation Project (http://rice.plantbiology.msu.edu/), respectively. The MADS-box protein sequences of *Medicago truncatula* were obtained from the LIS-Legume Information System (https://legumeinfo.org/home). Genome-wide transcriptome data of different alfalfa tissues were acquired from the CADL-Gene Expression Atlas (https://www.alfalfatoolbox.org). All transcriptome sequencing data used in this study are available in NCBI SRA (https://www.ncbi.nlm.nih.gov/sra/): SRR7091780-SRR7091794 (cold treatment) and SRR7160313-SRR7160357 (ABA, drought and salt treatments).

## Abbreviations

TFs: Transcription factors
Chr: Chromosome
1/2 MS: Half-strength murashige and skoog
ABA: Abscisic acid
FPKM: Fragments Per Kilobase of transcript per Million mapped reads

## References

[1] Messenguy F, Dubois E (2003). Role of MADS box proteins and their cofactors in combinatorial control of gene expression and cell development. Gene.

[2] Passmore S, Maine GT, Elble R, Christ C, Tye BK (1988). Saccharomyces cerevisiae protein involved in plasmid maintenance is necessary for mating of MAT alpha cells. J Mol Biol.

[3] Yanofsky MF, Ma H, Bowman JL, Drews GN, Feldmann KA, Meyerowitz EM (1990). The protein encoded by the *Arabidopsis* homeotic gene *agamous* resembles transcription factors. Nature.

[4] Sommer H, Beltrán JP, Huijser P, Pape H, Lönnig WE, Saedler H, Schwarz-Sommer Z (1990). *Deficiens*, a homeotic gene involved in the control of flower morphogenesis in *Antirrhinum majus*: the protein shows homology to transcription factors. EMBO J.

[5] Norman C, Runswick M, Pollock R, Treisman R (1988). Isolation and properties of cDNA clones encoding SRF, a transcription factor that binds to the c-*fos* serum response element. Cell.

[6] Alvarez-Buylla ER, Pelaz S, Liljegren SJ, Gold SE, Burgeff C, Ditta GS, de Pouplana LR, Martnez-Castilla L, Yanofsky MF (2000). An ancestral MADS-box gene duplication occurred before the divergence of plants and animals. Proc Natl Acad Sci USA.

[7] Smaczniak C, Immink RG, Angenent GC, Kaufmann K (2012). Developmental and evolutionary diversity of plant MADS-domain factors: insights from recent studies. Development.

[8] Henschel K, Kofuji R, Hasebe M, Saedler H, Münster T, Theissen G (2002). Two ancient classes of MIKC-type MADS-box genes are present in the moss *Physcomitrella patens*. Mol Biol Evol.

[9] Díaz-Riquelme J, Lijavetzky D, Martínez-Zapater JM, Carmona MJ (2009). Genome-wide analysis of MIKC^c^-type MADS box genes in grapevine. Plant Physiol.

[10] Theissen G, Kim JT, Saedler H (1996). Classification and phylogeny of the MADS-box multigene family suggest defined roles of MADS-box gene subfamilies in the morphological evolution of eukaryotes. J Mol Evol.

[11] Theissen G, Becker A, Di Rosa A, Kanno A, Kim JT, Münster T, Winter KU, Saedler H (2000). A short history of MADS-box genes in plants. Plant Mol Biol.

[12] Michaels SD, Ditta G, Gustafson-Brown C, Pelaz S, Yanofsky M, Amasino RM (2003). AGL24 acts as a promoter of flowering in *Arabidopsis* and is positively regulated by vernalization. Plant J.

[13] Dong TT, Hu ZL, Deng L, Wang Y, Zhu MK, Zhang JL, Chen GP (2013). A tomato MADS-box transcription factor, *SlMADS1*, acts as a negative regulator of fruit ripening. Plant Physiol.

[14] Lozano R, Angosto T, Gómez P, Payán C, Capel J, Huijser P, Salinas J, Martinez-Zapater JM (1998). Tomato flower abnormalities induced by low temperatures are associated with changes of expression of MADS-Box genes. Plant Physiol.

[15] Tardif G, Kane NA, Adam H, Labrie L, Major G, Gulick P, Sarhan F, Laliberté JF (2007). Interaction network of proteins associated with abiotic stress response and development in wheat. Plant Mol Biol.

[16] Lee S, Woo YM, Ryu SI, Shin YD, Kim WT, Park KY, Lee IJ, An G (2008). Further characterization of a rice AGL12 group MADS-box gene, *OsMADS26*. Plant Physiol.

[17] Khong GN, Pati PK, Richaud F, Parizot B, Bidzinski P, Mai CD, Bès M, Bourrié I, Meynard D, Beeckman T, Selvaraj MG, Manabu I, Genga AM, Brugidou C, Nang Do V, Guiderdoni E, Morel JB, Gantet P (2015). *OsMADS26* Negatively regulates resistance to pathogens and drought tolerance in rice. Plant Physiol.

[18] Agarwal P, Khurana P (2019). Overexpression of *TaMADS* from wheat promotes flowering by upregulating expression of floral promoters and provides protection against thermal stress. Plant Gene.

[19] Guo XH, Chen GP, Cui BL, Gao Q, Guo JE, Li AZ, Zhang LC, Hu ZL (2016). *Solanum lycopersicum* agamous-like MADS-box protein AGL15-like gene, *SlMBP11*, confers salt stress tolerance. Mol Breeding.

[20] Liu WX, Xiong CH, Yan LF, Zhang ZS, Ma LC, Wang YR, Liu YJ, Liu ZP. Transcriptome analyses reveal candidate genes potentially involved in Al stress response in alfalfa. Front Plant Sci. 2017;8:26. 10.3389/fpls.2017.00026 PMC5290290 28217130

[21] Chen HT, Zeng Y, Yang YZ, Huang LL, Tang BL, Zhang H, Hao F, Liu W, Li YH, Liu YB, Zhang XS, Zhang R, Zhang YS, Li YX, Wang K, He H, Wang ZK, Fan GY, Yang H, Bao AK, Shang ZH, Chen JH, Wang W, Qiu Q (2020). Allele-aware chromosome-level genome assembly and efficient transgene-free genome editing for the autotetraploid cultivated alfalfa. Nat Commun.

[22] Bao AK, Du BQ, Touil LL, Kang P, Wang QL, Wang SM (2016). Co-expression of tonoplast Cation/H(^+^) antiporter and H(^+^)-pyrophosphatase from xerophyte Zygophyllum xanthoxylum improves alfalfa plant growth under salinity, drought and field conditions. Plant Biotechnol J.

[23] Ashrafi E, Razmjoo J, Zahedi M, Pessarakli M (2014). Selecting alfalfa cultivars for salt tolerance based on some physiochemical traits. Agron J.

[24] Shen C, Du HL, Chen Z, Lu HW, Zhu FG, Chen H, Meng XZ, Liu QW, Liu P, Zheng LH, Li XX, Dong JL, Liang CZ, Wang T (2020). The chromosome-level genome sequence of the autotetraploid alfalfa and resequencing of core germplasms provide genomic resources for alfalfa research. Mol Plant.

[25] Genome-wide analysis (2018). of the MADS-box gene family in polyploid cotton (*Gossypium hirsutum*) and in its diploid parental species (*Gossypium arboreum* and *Gossypium raimondii*). Plant Physiol Bioch.

[26] Arora R, Agarwal P, Ray S, Singh AK, Singh VP, Tyagi AK (2007). MADS-box gene family in rice: genome-wide identification, organization and expression profiling during reproductive development and stress. BMC Genomics.

[27] Wang RZ, Ming ML, Li JM, Shi DQ, Qiao X, Li LT, Zhang SL, Wu J (2017). Genome-wide identification of the MADS-box transcription factor family in pear (*Pyrus bretschneideri*) reveals evolution and functional divergence. PeerJ.

[28] Parenicová L, de Folter S, Kieffer M, Horner DS, Favalli C, Busscher J, Cook HE, Ingram RM, Kater MM, Davies B, Angenent GC, Colombo L (2003). Molecular and phylogenetic analyses of the complete MADS-box transcription factor family in *Arabidopsis*: new openings to the MADS world. Plant Cell.

[29] Zhang ZB, Li HY, Zhang DF, Liu YH, Fu J, Shi YS, Song YC, Wang TY, Li Y (2012). Characterization and expression analysis of six MADS-box genes in maize (*Zea mays* L.). J Plant Physiol.

[30] Saha G, Park JI, Jung HJ, Ahmed NU, Kayum MA, Chung MY, Hur Y, Cho YG, Watanabe M, Nou IS (2015). Genome-wide identification and characterization of MADS-box family genes related to organ development and stress resistance in *Brassica rapa*. BMC Genomics.

[31] Zhou Q, Che TL, Wang YR, Liu ZP (2014). The development of 204 novel EST-SSRs and their use for genetic diversity analyses in cultivated alfalfa. Biochem Syst Ecol.

[32] Luo D, Wu YG, Liu J, Zhou Q, Liu WX, Wang YR, Yang QC, Wang ZY, Liu ZP (2018). Comparative transcriptomic and physiological analyses of *Medicago sativa* L. indicates that multiple regulatory networks are activated during continuous ABA treatment. Int J Mol Sci.

[33] Luo D, Zhou Q, Wu YG, Chai XT, Liu WX, Wang YR, Yang QC, Wang ZY, Liu ZP (2019). Full-length transcript sequencing and comparative transcriptomic analysis to evaluate the contribution of osmotic and ionic stress components towards salinity tolerance in the roots of cultivated alfalfa (*Medicago sativa* L.). BMC Plant Biol.

[34] Liu JY, Fu XD, Dong YW, Lu J, Ren M, Zhou NN, Wang CQ (2018). MIKC^c^-type MADS-box genes in *Rosa chinensis*: the remarkable expansion of ABCDE model genes and their roles in floral organogenesis. Hortic Res.

[35] Chen RG, Ma JH, Luo D, Hou XM, Ma F, Zhang YM, Meng YC, Zhang HF, Guo WL (2019). CaMADS, a MADS-box transcription factor from pepper, plays an important role in the response to cold, salt, and osmotic stress. Plant Sci.

[36] Ning K, Han YY, Chen ZJ, Luo C, Wang SL, Zhang WJ, Li L, Zhang XL, Fan SX, Wang Q (2019). Genome-wide analysis of MADS‐box family genes during flower development in lettuce. Plant Cell Environ.

[37] Tang W, Tu YY, Cheng XJ, Zhang LL, Meng HL, Zhao X, Zhang W, He B (2019). Genome-wide identification and expression profile of the MADS-box gene family in *Erigeron breviscapus*. PLoS One.

[38] Zhang YT, Tang DQ, Lin XC, Ding MQ, Tong ZK (2018). Genome-wide identification of MADS-box family genes in moso bamboo (*Phyllostachys edulis*) and a functional analysis of *PeMADS5* in flowering. BMC Plant Biol.

[39] Wang YS, Zhang JL, Hu ZL, Guo XH, Tian SB, Chen GP (2019). Genome-Wide Analysis of the MADS-Box Transcription Factor Family in *Solanum lycopersicum*. Int J Mol Sci.

[40] Yang SP, Gao JM, Wang LH, Sun XM, Xu PP, Zhang LW, Zhong QW (2019). Functional annotation and identification of MADS–box transcription factors related to tuber dormancy in *Helianthus tuberosus* L. 3 Biotech.

[41] Honma T, Goto K (2001). Complexes of MADS-box proteins are sufficient to convert leaves into floral organs. Nature.

[42] Nesi N, Debeaujon I, Jond C, Stewart AJ, Jenkins GI, Caboche M, Lepiniec L (2002). The *TRANSPARENT TESTA16* encodes the ARABIDOPSIS BSISTER MADS domain protein and is required for proper development and pigmentation of the seed coat. Plant Cell.

[43] Ren ZY, Yu DQ, Yang ZE, Li CF, Qanmber G, Li Y, Li J, Liu Z, Lu LL, Wang LL, Zhang H, Chen QJ, Li FG, Yang ZR (2017). Genome-wide identification of the MIKC-Type MADS-box gene family in *Gossypium hirsutum* L. unravels their roles in flowering. Front Plant Sci.

[44] Liu X, Sun ZC, Dong W, Wang ZJ, Zhang LS (2018). Expansion and functional divergence of the *SHORT VEGETATIVE PHASE (SVP)* genes in Eudicots. Genome Biol Evol.

[45] Zhou Q, Jia CL, Ma WX, Cui Y, Jin XY, Luo D, Min XY, Liu ZP (2019). MYB transcription factors in alfalfa (*Medicago sativa*): Genome-wide identification and expression analysis under abiotic stresses. Peer J.

[46] Mao P, Jin XY, Bao QY, Mei C, Zhou Q, Min XY, Liu ZP (2020). WRKY transcription factors in *Medicago sativa* L.: Genome-wide identification and expression analysis under abiotic stress. DNA Cell Biol.

[47] Cao B, Cui Y, Lou KK, Luo D, Liu ZP, Zhou Q (2020). Genome-wide identification and expression analysis of the *Dof* gene family in *Medicago sativa* L. under various abiotic stresses. DNA Cell Biol.

[48] Yamaguchi-Shinozaki K, Shinozaki K (2006). Transcriptional regulatory networks in cellular responses and tolerance to dehydration and cold stresses. Annu Rev Plant Biol.

[49] Tian Y, Dong QL, Ji ZR, Chi FM, Cong PH, Zhou ZS (2015). Genome-wide identification and analysis of the MADS-box gene family in apple. Gene.

[50] Wells CE, Vendramin E, Jimenez Tarodo S, Verde I, Bielenberg DG (2015). A genome-wide analysis of MADS-box genes in peach [*Prunus persica* (L.) Batsch]. BMC Plant Biol.

[51] Zhao HB, Jia HM, Wang Y, Wang GY, Zhou CC, Jia HJ, Gao ZS (2019). Genome-wide identification and analysis of the MADS-box gene family and its potential role in fruit development and ripening in red bayberry (*Morella rubra*). Gene.

[52] Tadege M, Sheldon CC, Helliwell CA, Upadhyaya NM, Dennis ES, Peacock WJ (2003). Reciprocal control of flowering time by *OsSOC1* in transgenic *Arabidopsis* and by *FLC* in transgenic rice. Plant Biotechnol J.

[53] Shitsukawa N, Ikari C, Mitsuya T, Sakiyama T, Ishikawa A, Takumi S, Murai K (2007). Wheat *SOC1* functions independently of *WAP1/VRN1*, an integrator of vernalization and photoperiod flowering promotion pathways. Physiol Plant..

[54] Zhong XF, Dai X, Xu JH, Wu HY, Liu B, Li HY (2012). Cloning and expression analysis of *GmGAL1*, *SOC1* homolog gene in soybean. Mol Biol Rep.

[55] Bai G, Yang DH, Cao PJ, Yao H, Zhang YH, Chen XJ, Xiao BG, Li F, Wang ZY, Yang J, Xie H (2019). Genome-Wide Identification, Gene structure and expression analysis of the MADS-Box gene family indicate their function in the development of Tobacco (*Nicotiana tabacum* L.). Int J Mol Sci.

[56] Kaufmann K, Melzer R, Theissen G (2005). MIKC-type MADS domain proteins: structural modularity, protein interactions and network evolution in land plants. Gene.

[57] Jia JT, Zhao PC, Cheng LQ, Yuan GX, Yang WG, Liu S, Chen SY, Qi DM, Liu GS, Li XX (2018). MADS-box family genes in sheepgrass and their involvement in abiotic stress responses. BMC Plant Biol.

[58] Yang F, Xu F, Wang XH, Liao YL, Chen QW, Meng XX (2016). Characterization and functional analysis of a MADS-box transcription factor gene (*GbMADS9*) from *Ginkgo biloba*. Sci Hortic.

[59] Cao J, Sun L, Li J, Zhou C, Cheng L, Chen K, Yan B, Qian W, Ma Q, Duan W (2018). A novel three-miRNA signature predicts survival in cholangiocarcinoma based on RNA-seq data. Oncol Rep.

[60] Zhang T, Chen H, Qi L, Zhang J, Wu R, Zhang Y, Sun Y (2018). Transcript profiling identifies early response genes against FMDV infection in PK-15 cells. Viruses..

[61] Zhang XD, Fatima M, Zhou P, Ma Q, Ming R (2020). Analysis of MADS-box genes revealed modified flowering gene network and diurnal expression in pineapple. BMC Genomics.

[62] Gotz S, Garcia-Gomez JM, Terol J, Williams TD, Nagaraj SH, Nueda MJ, Robles M, Talon M, Dopazo J, Conesa A (2008). High-throughput functional annotation and data mining with the Blast2GO suite. Nucleic Acids Res.

[63] Chen CJ, Chen H, Zhang Y, Thomas HR, Frank MH, He YH, Xia R (2020). TBtools: an integrative toolkit developed for interactive analyses of big biological data. Mol Plant.

[64] O’Rourke JA, Fu F, Bucciarelli B, Yang SS, Samac DA, Lamb JAFS, Monteros MJ, Graham MA, Gronwald JW, Krom N, Li J, Dai XB, Zhao PX, Vance CP (2015). The *Medicago sativa* gene index 1.2: a web-accessible gene expression atlas for investigating expression differences between *Medicago sativa* subspecies. BMC Genomics.

[65] Camacho C, Coulouris G, Avagyan V, Ma N, Papadopoulos J, Bealer K, Madden TL (2009). BLAST+: architecture and applications. BMC Bioinformatics.

[66] Livak KJ, Schmittgen TD (2001). Analysis of relative gene expression data using real-time quantitative PCR and the 2^–∆∆CT^ method. Methods.

