# Supplementary material for: Genome-wide identification of the MADS-box transcription factor family in autotetraploid cultivated alfalfa (Medicago sativa L.) and expression analysis under abiotic stress
Source: BMC Genomics. 2021 Aug 7;22:603. doi: 10.1186/s12864-021-07911-9 (PMC8348820; doi:10.1186/s12864-021-07911-9)
Supplement: Supplementary file 2 — Additional file 2: Figure S1. Phylogenetic tree of Type II MADS-box genes in alfalfa and Arabidopsis constructed using the NJ method in MEGA-X. Group II is divided into 13 subgroups represented by different colors, and alfalfa and Arabidopsis MADS-box genes are indicated by blue stars and red triangles, respectively. Figure S2. Gene structure analysis of MsMADS-box genes in alfalfa. Black lines represent the introns, and green blocks represent the exons. Figure S3. Motif analysis of MsMADS-box proteins. Each motif is represented by boxes of different colors for motifs 01 to 20. Figure S4. Cis-regulatory elements analysis of the promoter regions of MADS-box genes of alfalfa. The differently colored boxes with numbers indicate the numbers of cis-regulatory elements in the promoter regions of MADS-box genes. Figure S5. Expression levels of 104 MADS-box genes in response to cold treatment in alfalfa. Heat map showing the changes in the relative expression of these genes at 0 (CK), 2, 6, 24, and 48 h under cold treatment at 4 ℃ in the whole seedling. Groups A to F exhibited six expression patterns of the tested MADS-box genes under cold treatment. Figure S6. Expression levels of 104 MADS-box genes in alfalfa under ABA, drought, and salt treatments. Heatmap showing the relative expression levels of total MADS-box genes at different time points after ABA treatment (0, 1, 3 and 12 h), drought treatment (0, 1, 3, 6, 12, and 24 h 1 h and 12 h after removal), and salt treatment (0, 1, 3, 6, 12, and 24 h and 1 h and 12 h after removal) in the root tip; “CK” represents 0 h. Groups A to I show nine expression patterns of MADS-box genes under the three treatments. Figure S7. Gene expression analysis of eight MsMADS-box genes without cold treatment for 0, 2, 6, 24, and 48 h using qRT-PCR. The error bars indicate the standard errors of three biological replicates. Asterisks represent significant differences compared with “CK”, and P < 0.05 (*) was considered highly significant. Figu [file 12864_2021_7911_MOESM2_ESM.docx]

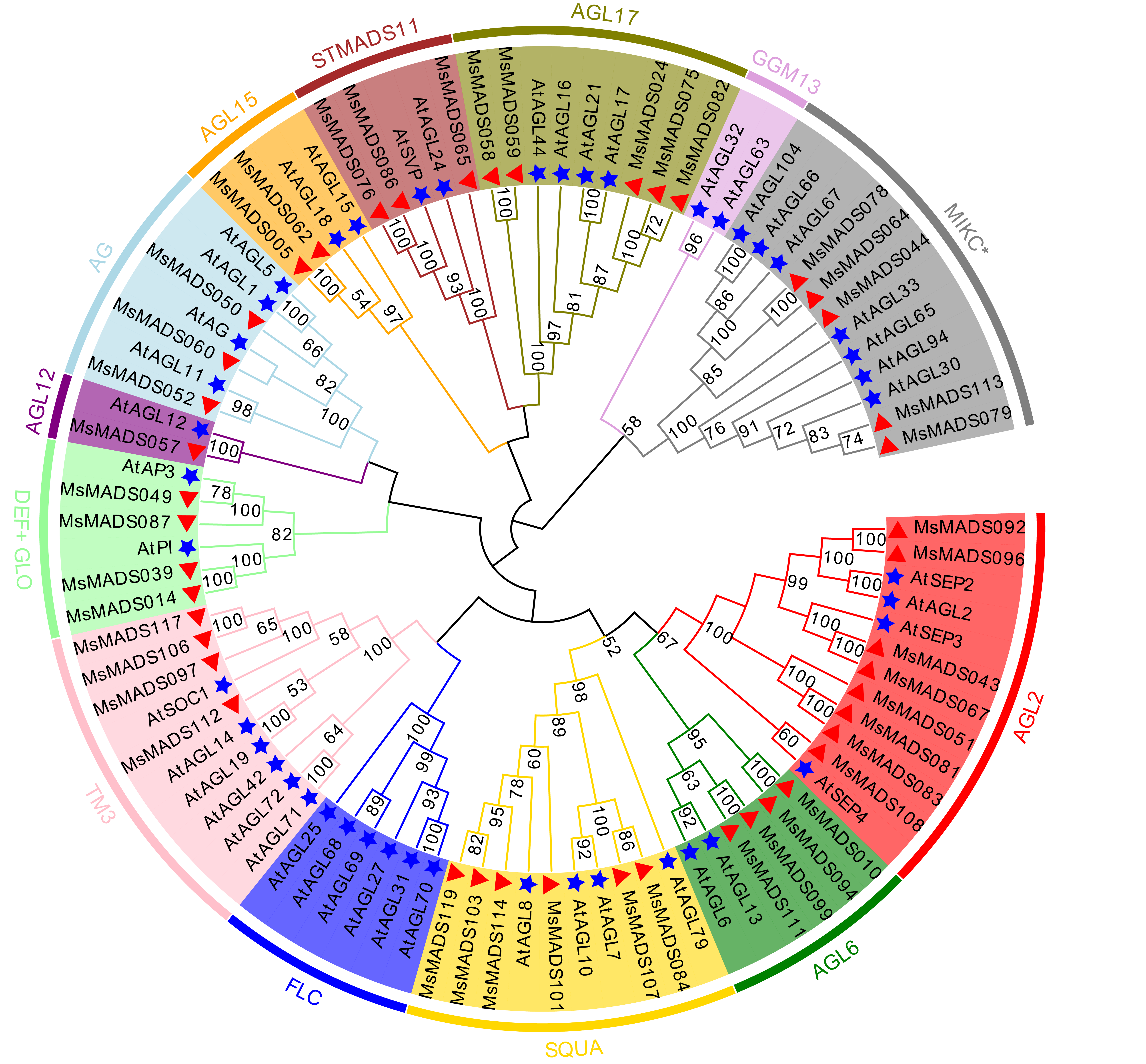


Fig. S1 Phylogenetic tree of Type II *MADS-box* genes in alfalfa and *Arabidopsis* constructed using the NJ method in MEGA-X. Group II is divided into 13 subgroups represented by different colors, and alfalfa and *Arabidopsis MADS-box* genes are indicated by blue stars and red triangles, respectively.


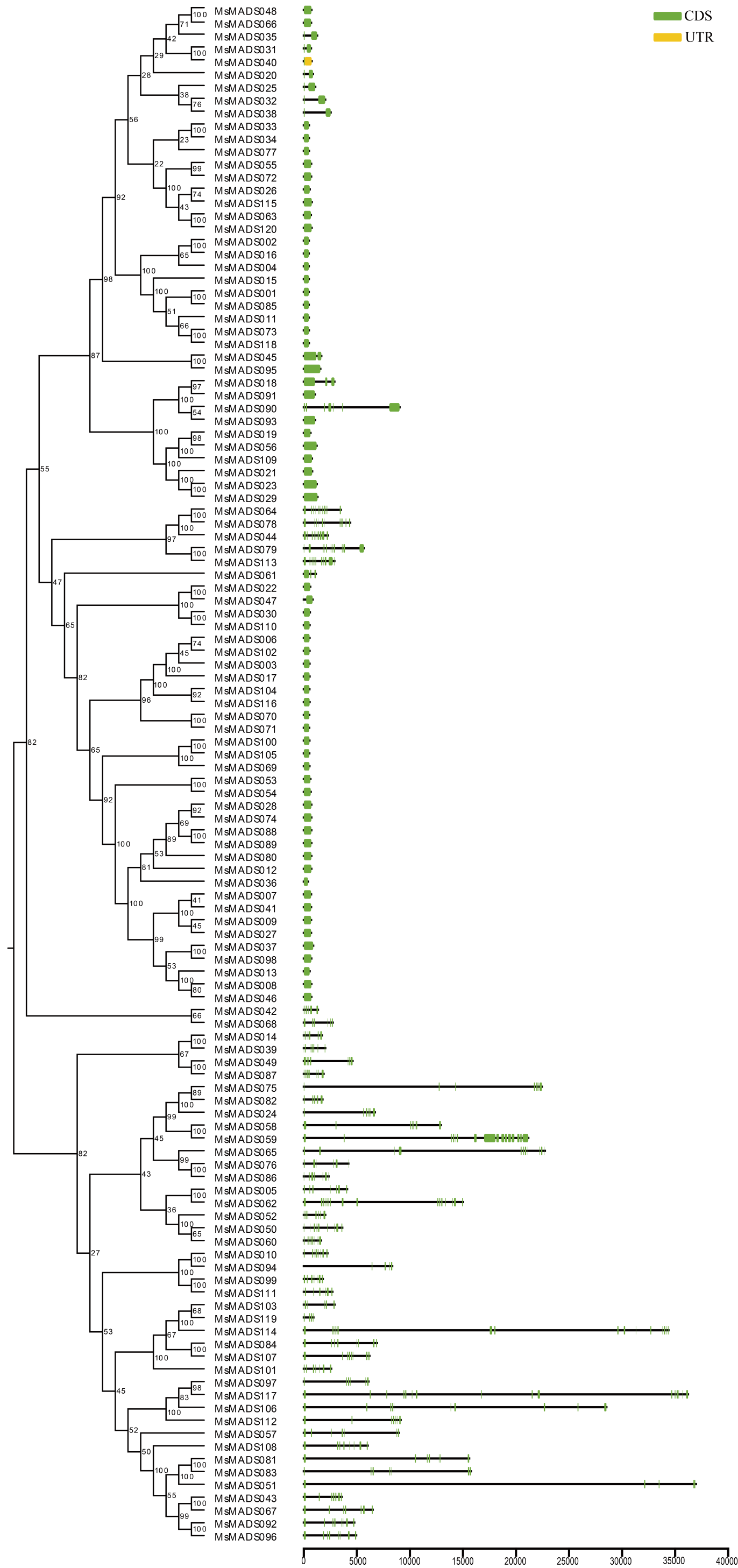


Fig. S2 Gene structure analysis of *MsMADS-box* genes in alfalfa. Black lines represent the introns, and green blocks represent the exons.


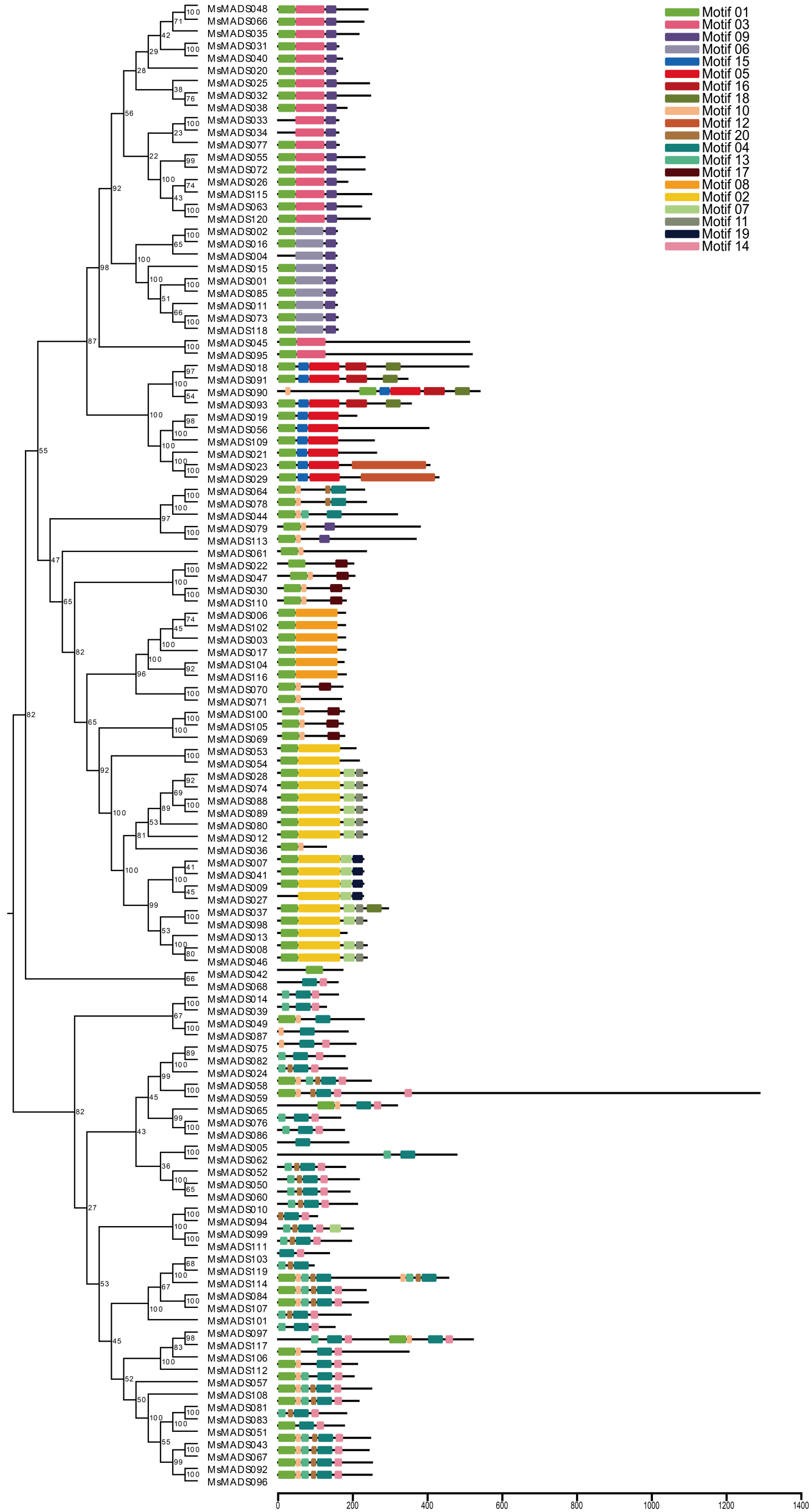


Fig. S3 Motif analysis of MsMADS-box proteins. Each motif is represented by boxes of different colors for motifs 01 to 20.


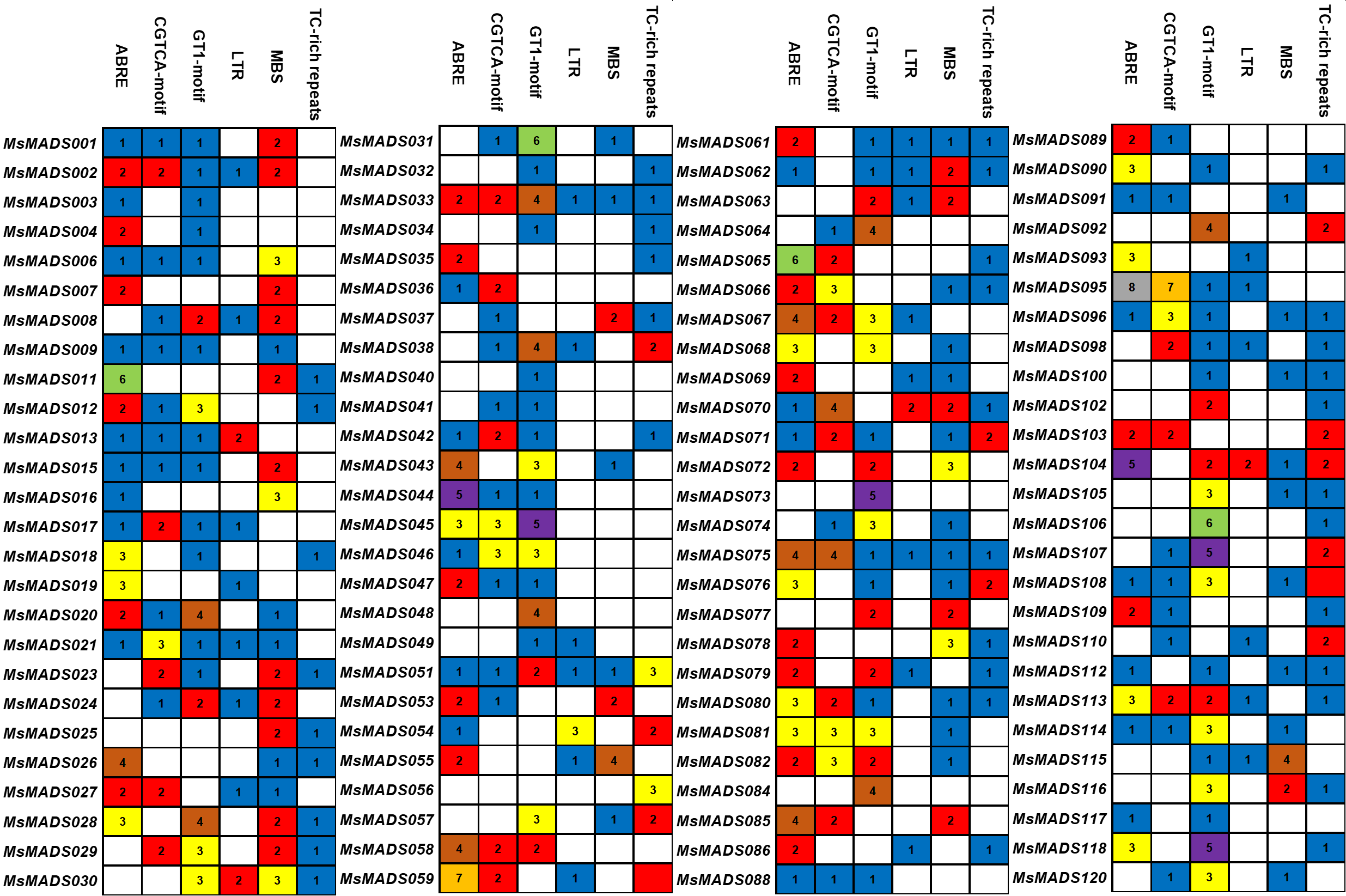
 Fig. S4 *Cis*-regulatory elements analysis of the promoter regions of *MADS-box* genes of alfalfa. The differently colored boxes with numbers indicate the numbers of *cis*-regulatory elements in the promoter regions of *MADS-box* genes.


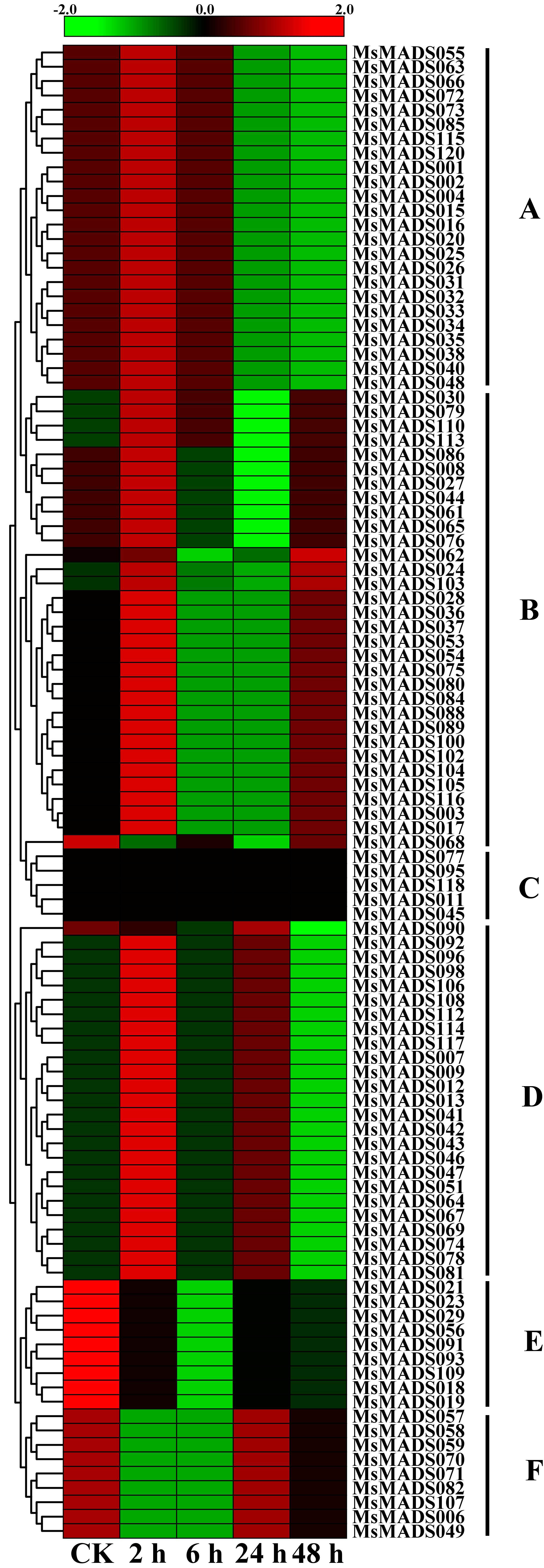


Fig. S5 Expression levels of 104 *MADS-box* genes in response to cold treatment in alfalfa. Heat map showing the changes in the relative expression of these genes at 0 (CK), 2, 6, 24, and 48 h under cold treatment at 4 ℃ in the whole seedling. Groups A to F exhibited six expression patterns of the tested *MADS-box* genes under cold treatment.


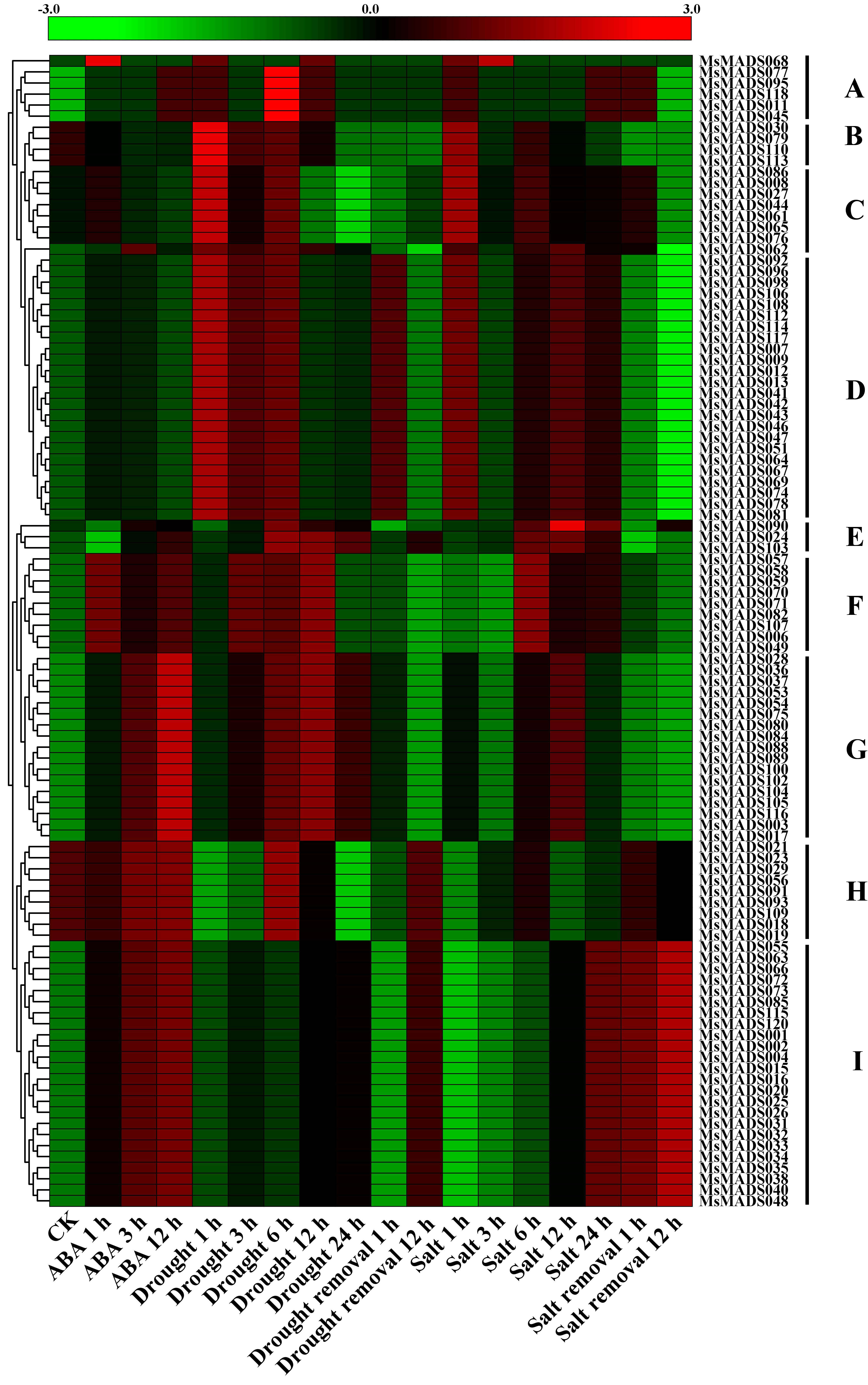


Fig. S6 Expression levels of 104 *MADS-box* genes in alfalfa under ABA, drought, and salt treatments. Heatmap showing the relative expression levels of total *MADS-box* genes at different time points after ABA treatment (0, 1, 3 and 12 h), drought treatment (0, 1, 3, 6, 12, and 24 h 1 h and 12 h after removal), and salt treatment (0, 1, 3, 6, 12, and 24 h and 1 h and 12 h after removal) in the root tip; “CK” represents 0 h. Groups A to I show nine expression patterns of *MADS-box* genes under the three treatments.


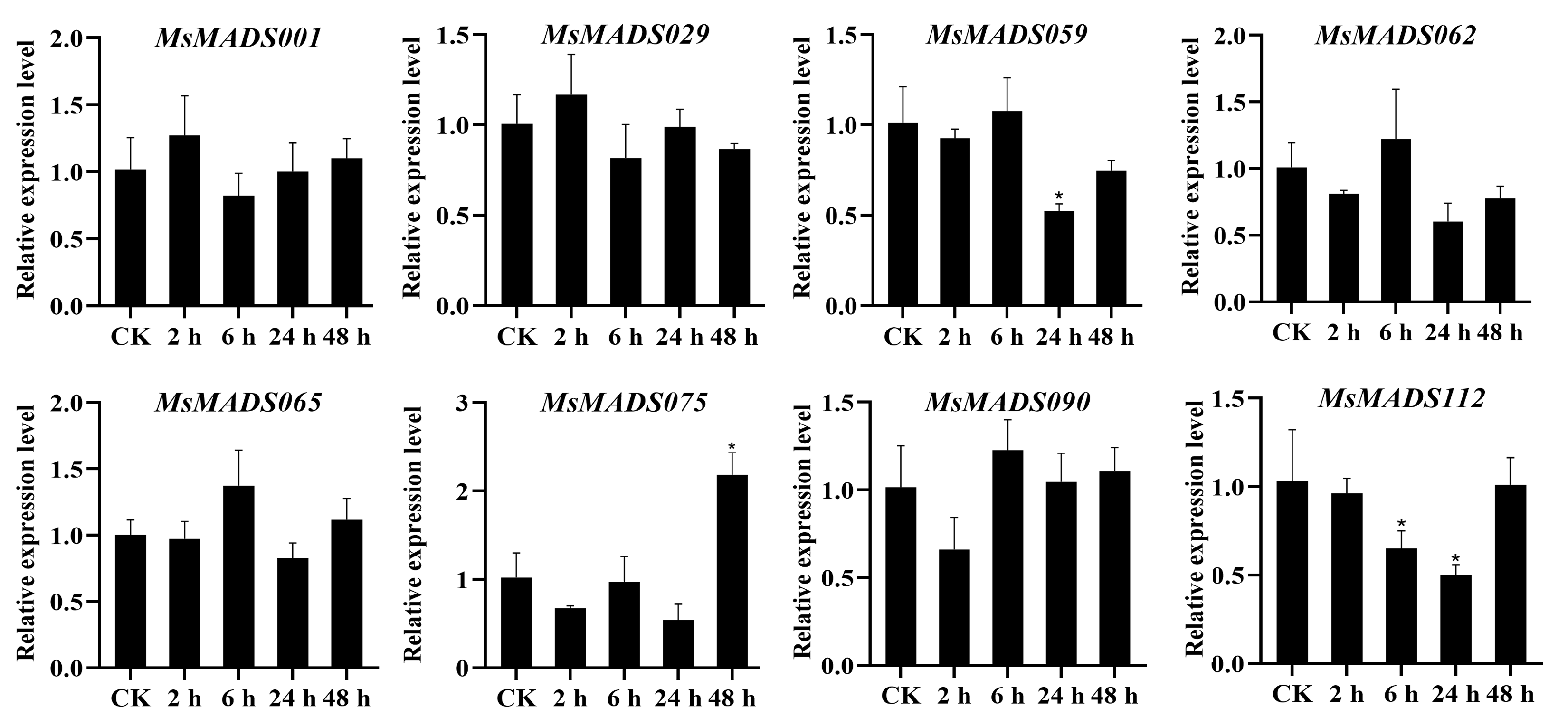


Fig. S7 Gene expression analysis of eight *MsMADS-box* genes without cold treatment for 0, 2, 6, 24, and 48 h using qRT-PCR. The error bars indicate the standard errors of three biological replicates. Asterisks represent significant differences compared with “CK”, and P < 0.05 (^*^) was considered highly significant.


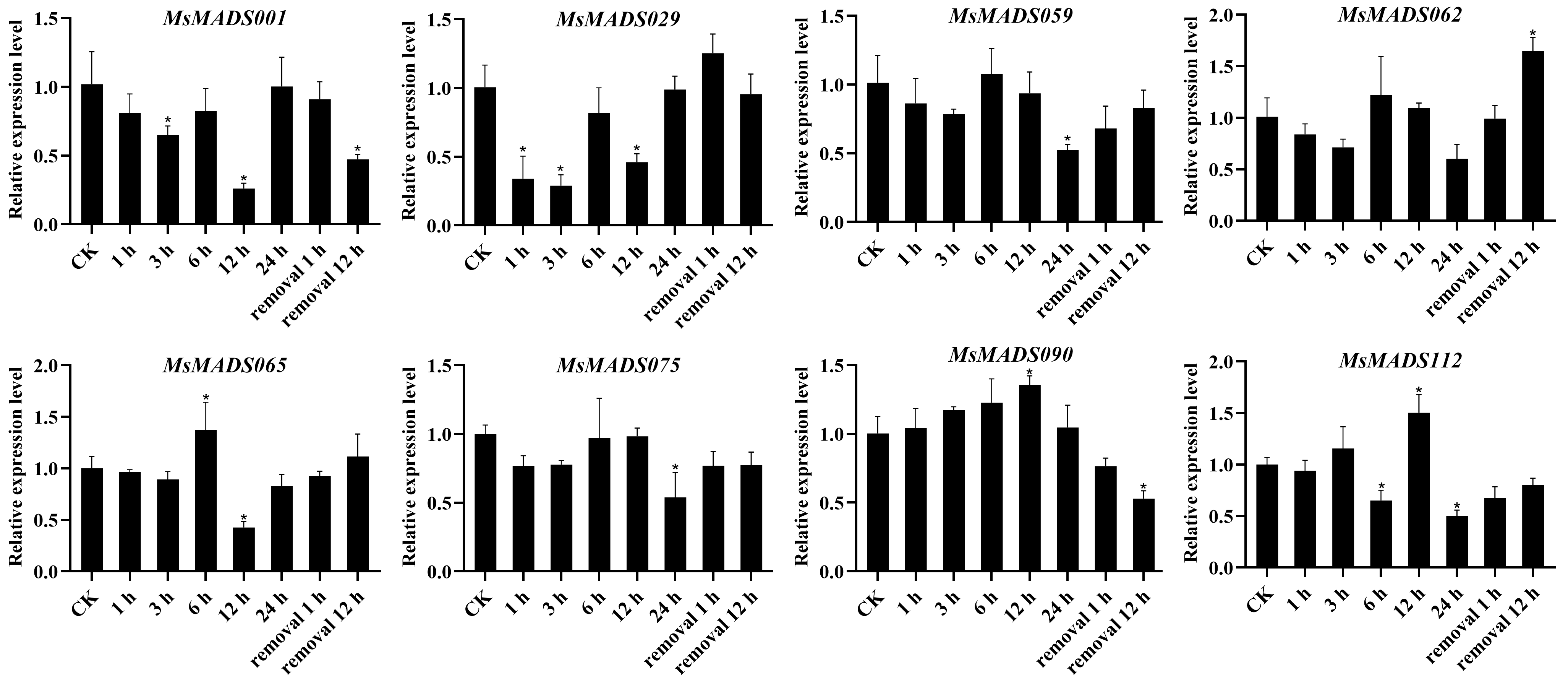
 Fig. S8 Gene expression analysis of eight *MsMADS-box* genes without drought and salt treatment for 0, 1, 3, 6, 12, and 24 h and 1 h and 12 h after removal using qRT-PCR. The error bars indicate the standard errors of three biological replicates. Asterisks represent significant differences compared with “CK”, and P < 0.05 (^*^) was considered highly significant.


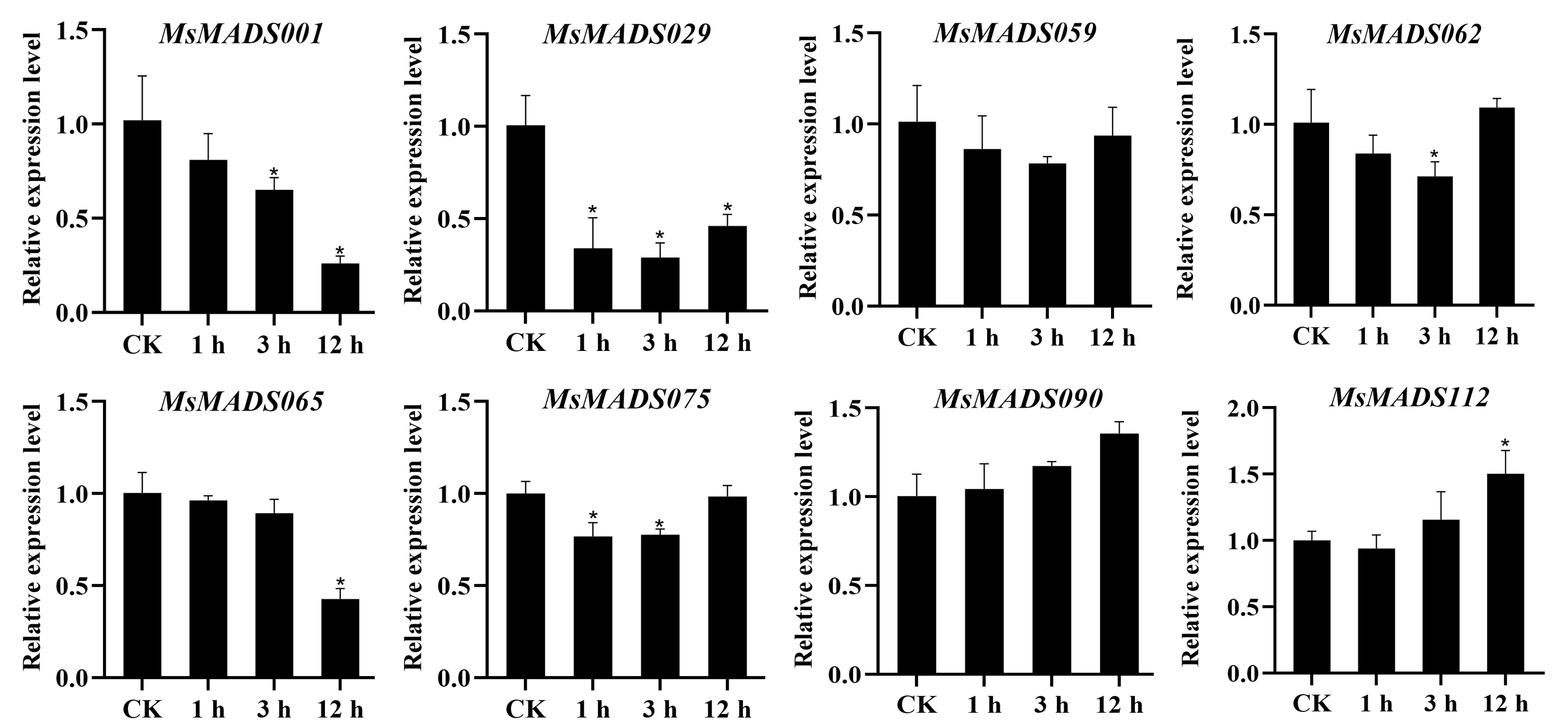
 Fig. S9 Gene expression analysis of eight *MsMADS-box* genes without ABA treatment for 0, 1, 3, and 12 h using qRT-PCR. The error bars indicate the standard errors of three biological replicates. Asterisks represent significant differences compared with “CK”, and P < 0.05(^*^) was considered highly significant.
